# Research Progress on Heavy Metals Pollution in the Soil of Smelting Sites in China

**DOI:** 10.3390/toxics10050231

**Published:** 2022-04-30

**Authors:** Muhammad Adnan, Baohua Xiao, Peiwen Xiao, Peng Zhao, Ruolan Li, Shaheen Bibi

**Affiliations:** 1 State Key Laboratory of Environmental Geochemistry, Institute of Geochemistry, Chinese Academy of Sciences, Guiyang 550081, China; adnan@mail.gyig.ac.cn (M.A.); xiaopeiwen@mail.gyig.ac.cn (P.X.); zhaopeng@mail.gyig.ac.cn (P.Z.); liruolan@mail.gyig.ac.cn (R.L.); 2 University of Chinese Academy of Sciences, Beijing 100049, China; 3 Lanzhou Veterinary Research Institute, Chinese Academy of Agricultural Sciences, Lanzhou 730046, China; shaheenwazir12@gmail.com; 4 Graduate School, Chinese Academy of Agricultural Sciences, Beijing 100081, China

**Keywords:** heavy metal, pollution, soil, smelting site, hydrometallurgy, resource recovery, remediation

## Abstract

Contamination by heavy metals is a significant issue worldwide. In recent decades, soil heavy metals pollutants in China had adverse impacts on soil quality and threatened food security and human health. Anthropogenic inputs mainly generate heavy metal contamination in China. In this review, the approaches were used in these investigations, focusing on geochemical strategies and metal isotope methods, particularly useful for determining the pathway of mining and smelting derived pollution in the soil. Our findings indicate that heavy metal distribution substantially impacts topsoils around mining and smelting sites, which release massive amounts of heavy metals into the environment. Furthermore, heavy metal contamination and related hazards posed by Pb, Cd, As, and Hg are more severe to plants, soil organisms, and humans. It’s worth observing that kids are particularly vulnerable to Pb toxicity. And this review also provides novel approaches to control and reduce the impacts of heavy metal pollution. Hydrometallurgy offers a potential method for extracting metals and removing potentially harmful heavy metals from waste to reduce pollution. However, environmentally friendly remediation of contaminated sites is a significant challenge. This paper also evaluates current technological advancements in the remediation of polluted soil, such as stabilization/solidification, natural attenuation, electrokinetic remediation, soil washing, and phytoremediation. The ability of biological approaches, especially phytoremediation, is cost-effective and favorable to the environment.

## 1. Introduction

In many parts of the world, heavy metal pollution is currently a major issue [1,2]. Chemically, heavy metal elements have an atomic mass greater than 20 and a gravity greater than 5 g·cm^−3^ [3,4,5], including Mercury (Hg), Cadmium (Cd), Copper (Cu), Nickel (Ni), Lead (Pb), Arsenic (As), Chromium (Cr), and Zinc (Zn) [6]. Metalloid arsenic is frequently placed in the heavy metal sort due to chemical characteristics and environmental response similarities [7,8]. Since industrialization and technological development emerged, heavy metal poisoning of farming soil has been a significant concern in China [9]. According to the State Environmental Protection Administration, China has severe soil metal pollution [10]. Heavy metal have attracted certain attention from environmental chemists among all the contaminants due to their persistence and toxicity.

Over the last few decades, the soil has been polluted by toxic hazard materials worldwide, which has become a significant source of concern [11,12]. Soil hazard elements are difficult to break down and can enter food and water supply systems, resulting in long-term damage to food protection and human wellbeing [13,14]. Heavy metals have been classified as priority control contaminants by the USEPA (United States Environmental Protection Agency), and it has attracted increasing attention throughout many regions of the world due to their potentially hazardous, chronic, and irreversible features [15,16]. While Cu is a necessary trace element, excessive amounts can harm health [17]. According to two current Chinese government ministry surveys, after Cd, Pb is the considerable widespread heavy metal in the environment [18]. In China, the foremost National Soil Pollution Investigation reported that 1.50 percent of soil samples were contaminated with Pb between 2005 and 2013 [19,20]. Pb exposure to humans occurs mainly from direct intake of dust and soil via hand-to-mouth contact, particularly among children [21]. Especially in developing nations, blood (Pb) toxicity is a major public health concern [22]. Kids living near the smelters have high blood lead levels (BLLs) [23].

Heavy metals reach the environment in numerous anthropogenic activities such as mine waste, smelting, metallurgical and chemical, and natural processes [24,25,26]. Lithogenesis, weathering, erosion, and other geological processes are primarily responsible for natural sources [24]. In China, the major causes of heavy metals contamination are anthropogenic sources, including industrial pollution from non-ferrous mining and smelting operations [27]. Numerous non-ferrous metal mines have been extensively exploited throughout China during the previous three decades. Heavy metal contamination has been causing numerous environmental hazards [28], including water, atmosphere, and soil. The addition of heavy metals in soils from several Chinese towns revealed that many of them were contaminated with multiple types of heavy metals [29]. Industrialization in China has increased heavy metal pollution in the soil and water. Anthropogenic inputs mainly cause heavy metal contamination in China [27], including industrial discharges from mining and non-ferrous smelting operations [8,30,31,32,33,34]. China has the fastest growing economy and largest developing nation in the world. China has surpassed the world’s most significant raw and refined lead manufacturer and is the world’s leading consumer [18].

In recent decades, central and south China has become one of the country’s fastest-growing regions. Manufacturing and farming activities, community growth, and the widespread usage of chemicals are linked to severe contamination issues in this area [35]. The mining and smelting of nonferrous metals are two major sources of heavy metal pollution in soils. Some farmlands near smelting/mining activities become polluted [36]. For instance, Pb/Zn mining and smelting have resulted in trace elements like As, Cd, and Pb being found in soil and river sediments in Hunan, Guizhou, Guangdong, and Yunnan [19,37,38]. Since the early 1920s, Pb pollution has been found in China [39].

Nevertheless, in recent decades, Henan Province in China has overcome other areas to become the country’s leading manufacturer. Despite extensive research in the literature on soil pollution by heavy metals near China’s Pb/Zn mining and smelting locations [23]. In China, around 82% of the polluted farming soils include toxic inorganic contaminants, including Pb, Cd, Cr, and As [40]. Prerequisites for soil pollution precluding and control include understanding the factors of heavy metal pollution in soil and the recognition of environmental exposure hazards [41] and give significant knowledge when forming judgments about remediating polluted soils [7]. The earlier we create and performed measures to prevent or reduce this type of contamination [41]. The prevention of soils contaminated by heavy metals is necessary to control sources and advance polluted soils’ remediation. This review aims to study the occurrences and distribution patterns of heavy metals in soil pollution in Chinese mining regions and assess the health risks caused by these polluted soils. The review also emphasizes the remediation mechanisms and efficacy of emerging technologies and crucial future research areas in this field.

## 2. Investigational Methods

### 2.1. Studies of Geochemical Analysis

Reviewing the relevant literature collected during the past few years, this studies soil heavy metal concentrations in China, especially in mining and smelting areas, where large-scale heavy metal pollution intrusions are widespread. This study included heavy metals including Cr, Cd, Ni, Cu, Ag, Hg, Zn, As, and Pb. According to the USEPA, they are all priority heavy metal contaminants. Data were collected from the main literature databases such as Elsevier Science Direct, Science Online, Web of Science, and China national knowledge infrastructure (CNKI).

In terms of spatial distribution, heavy metal concentrations were mainly found near smelter areas in China. Soil samples from (0–20 cm) deep were taken from non-ferrous smelting sites in 19 main provinces to examine the distribution of heavy metals in soil [41]. Due to the distribution characteristics of heavy metals in soil, it is required to consider a special sampling technique near human-affected areas [42]. Conventional sampling approaches have relied on the collection of soil samples near mines [8]. Smelter emissions significantly impact topsoils, the most vulnerable part of the environment [43].

Pb-smelteries might be found in numerous Chinese provinces. According to the [44] study, tailings, smelter slags, and soil from mining sites had the least Pb uptake. However, [45] showed that the Pb content in surface soil samples reduced significantly with space from the Pb smelter, from about 1500 mg/kg at locations less than 2 km from the smelter to about 100 mg/kg at locations more than 2 km from the smelter [46], found that 89.3% of the samples in the smelting site surpassed the standard’s threshold soil values. For instance, China’s four largest industrial regions are the (Beijing-Tianjin-Tangshan zone), (Central and South Liaoning zones), (Yangtze River Delta zone), and (Pearl River Delta zone) [47].

Another sampling approach involves collecting soil profiles at different ranges from the smelter stack and orienting it to the dominant wind path (upwind vs. downwind) [48]. However, other investigations have collected only a few top centimeters (0–5 cm) [48]. Generally, Topsoil samples were obtained at deeps of (0–10 cm) [49], (0–15 cm) [50], and (0–20 cm) [50,51,52,53,54]. In 2009 [52] took six soil profiles sampled from 70-cm depths in forested and prairie sites at varied ranges from the (Cu) smelting plant, and the soil cores were typically 60, 80, or 100 cm deep [50]. However, most previous research concentrated on a subset of normal regions, including chemical plants, mines, and smelting enterprises, to detect heavy metal concentrations at sampling locations and subsequently evaluate pollution levels and ecological hazards [55].

Throughout the investigations, numerous approaches were utilized to detect the levels of heavy metals in soil samples. Still, the most frequent approaches are atomic absorption spectroscopy (AAS) [41], inductively coupled plasma-mass spectrometry (ICP-MS) [41,51,54,56], and [50] were used Inductively coupled plasma atomic emission spectroscopy (ICP-AES), instruments to determine total Pb amounts in digestion solutions. ICP-MS technology enables greater analytical accuracy and minimizes interfering signals. And ICP-MS offers many unique features and has been successfully applied to environmental monitoring, but it is essential to understand and master the precautions. For ICP-AES and ICP-MS, the researcher should be aware of their instrument’s properties (acidity, organic content, etc.), improving its stability during measurement and reducing maintenance frequency. The instrument’s stability relies on the stability of the test environment. The scientific community widely accepted the sampling tactics and data analytical techniques utilized in the selected studies.

### 2.2. Application of Isotopes

With the fast advancement of instrumental analysis techniques, metal isotope techniques to track the origin and distribution of heavy metals in the environment have become widespread [54]. Since the late 1990s, the MC-ICP-MS (multi-collector inductively coupled plasma mass spectrometer) has been offered an alternative to the TIMS for metal isotope analysis. It has evolved into the most commonly used technology in metal stable isotope research in the twenty-first century [57]. Isotope ratio analysis (IRA) is a useful approach for determining metal “fingerprints” in environmental samples [57]. Whereas the radioactive isotopes decompose into daughter isotopes of other elements, stable isotopes were generated during the big bang and have remained “stable” [57]. In certain circumstances, stable isotopes can be used to trace or identify sources [58,59]. In nature, Pb exists in four stable isotopes: 204Pb (1.4%), 206Pb (24.1%), 207Pb (22.1%) and 208Pb (52.4%) [57]. The broad range of isotopic abundance ratios between the four stable isotopes of Pb is found in various ore deposits throughout the globe. Pb isotopes are the commonly used and prosperous method for investigating the sources and mobility of heavy metals in environmental samples, including soils, sediments, water systems, and the atmosphere [54]. The proportions of Pb isotopes in different fractions generated by selective leaching can be used to determine whether the portion of smelter-derived Pb is potentially mobile [48]. Furthermore, ref. [45] show that the Pb isotope ratios revealed that anthropogenic Pb impacted the upper soil horizons.

According to the review by [60], only two high-quality Pb isotope records have been reconstructed from Chinese peatlands, one of which is the Motianling peatland in NE China, while other records are scattered across China. However, ref. [43] notes that some topsoil samples show a mixture of smelter-derived Pb (predominant) and petrol-derived Pb in their Pb isotopic composition (minor contribution). Lead ores of various geological epochs result in systematic isotopic variation amongst refineries [58]. The use of stable Pb isotopes to distinguish man-made Pb from natural Pb generated from mineral weathering is a valuable tool [61]. Pb isotope tracing indicated that mining and smelting enterprises were the primary origins of Pb in atmospheric dustfall and farming soils. Thus, while diesel transportation discharges were the primary origin of Pb in plants, atmospheric deposition was a significant source of Pb for plants [26]. Plant root water uptake patterns can be assessed using the stable isotope tracing approach, both effective and non-invasive. Also, no isotope fractionation happens during water passage from the soil to the plant [62]. Likewise, plant roots absorb (Hg) from soils, affecting soil (Hg) accessibility and stabilizing (Hg) under the soil surface (a process referred to as phytostabilization); additionally, investigations of stable (Hg) in roots have revealed diverse (Hg) sources [63].

Compared to Beijing and Shanghai soils, the Pb isotope fingerprints from Haikou were all significantly different but identical to those from Guangzhou soils, implying that Pb originates from a common source throughout southern China cities [29]. Moreover, rock weathering contributes significantly to soil (Cd) deposition, particularly in SW China [57]. It was illustrated by (Wang, Jin, et al. 2020), showing that Cd isotope compositions of farming soils sampled from the Jianghan Plain, China, were identical to those in smelter dust and furnace fly ash, showing that manufacturing sources including smelting and refining activities are the primary origin of Cd. Recently, did not discover any Cd fractionation during the smelting process itself but proposed that the interaction of Cd with solid soil components after it deposition in soil could be responsible for the differences reported in different strata of forest soils, and Zn isotopes also showed varying effects [48]. The average origin contribution of atmospheric Hg(0) deposition in surface soils in Tibetan wetlands in China is 26% [63], and atmospheric accumulation is frequently recognized as a leading origin of (Hg) due to its elevated volatility [57]. Cu and Zn from smelters can be detected in the soil through the natural abundance of stable metal isotopes, which can be used to distinguish anthropogenic and natural Cu and Zn in the soil and may assist in clarifying the metal’s distribution in the soil [64]. Chromium has four stable isotopes with masses of 50 (4.35%), 52 (83.8%), 53 (9.50%), and 54 (2.37%) [65]. Thus, stable Cr isotope ratios can be utilized to define the extent of Cr(VI) reduction in groundwater [65]. Zn has five stable isotopes with masses of 64, 66, 67, 68, and 70, with an average enormous amount of (48.63%), (27.90%), (4.10%), (18.75%), and (0.62%), respectively [66]. Zn isotopes of natural resources on Earth have minor fluctuations but become significant at elevated temperatures, so Zn isotopes are particularly helpful in identifying contamination caused by smelters [64]. It was surprising that the hydrometallurgical effluent and the fume were heavier isotopically than the Zn ore concentrate in the Trial Pb-Zn smelting operation (B.C., Canada) because of the Zn isotope fractionation [48]. Ref. [67] found no similar notable isotopic differences in any solid material or crucial reaction results throughout the analyzed metallurgical process. Copper stable isotopes, namely 63Cu (69.17%) and 65Cu (30.83%) [66]. There are five stable isotopes of nickel (Ni), namely ^58^Ni, ^60^Ni, ^61^Ni, ^62^Ni, and ^64^Ni, with particular natural abundances of 68.08%, 26.22%, 1.14%, 3.63%, and 0.93% [68]. Cu stable isotope composition is broader than Ni, with δ65Cu of −16.5‰ to +10‰ in minerals and ores. Sediments, secondary ore minerals, biological material, and mine tailings have significant variations in δ65Cu [69]. Moreover, numerous ways are employed to trace pollution from mining and smelting, but stable Pb isotope has revealed its effectiveness and will continue to be useful in tracing contamination.

## 3. Evaluation of Heavy Metal Pollution

### 3.1. Mechanism of Heavy Metal Pollution

Heavy metals can be found in various forms in both water and soil [70]. The natural sources include precipitation, weathering, corrosion, and volcanic; Man-made sources include mining, smelting, electroplating, pesticide and (phosphate) fertilizer use, agricultural biosolids, sludge discharge, and industrial combustion, and so on [2]. As a result of urbanization processes, not only fundamental qualities of the impacted soils were changed (such as pH and texture), but also cation exchange capacity and bulk density were decreased, and dangerous compounds such as heavy metals were unintentionally deposited in the soils [71]. Soil organic carbon and cation exchange capacity were the main soil characteristics that determined the chemical deposits and the potential to sorb metal from urban soils [71]. Soil organic matter (SOM) is a term that refers to all non-living organic compounds found in soil. SOM serves as a vital proxy for soil properties since it aggregates the major particles in the soil and plays a critical role in stabilizing the soil’s physical structure [72]. The bulk of SOM comprises humic substances, complex combinations of naturally occurring organic macromolecules generated during the biological and abiotic alterations of soil-plant, animal wastes, and, microbial [73]. A large quantity of incoming Pb is absorbed on SOM surfaces; however, following degradation/decomposition, a larger amount of Pb is transferred to pedogenic birnessite and ferrihydrite surfaces [74]. Consequently, heavy metal sorption is caused by numerous soil components, including humic substances, phyllosilicates, carbonates, variable charge minerals, and microbes [75]. Bacteria have particular genetic pathways and play a key role in environmental pollution reduction [76]. Soil bacteria are challenging to examine, even though they are the fundamental players in the biosphere’s health. They transform plant biomass into soil organic matter. However, soil habitats are incredibly dynamic and structurally complex. Because the internal structure of the soil is very complex, the existing approaches for determining the living behavior of soil microorganisms are failed.

Although the mechanisms/methods for the elimination of heavy metals are numerous, they can be organized into one sort: non-destructive mechanisms like adsorption, ion exchange, surface complexation, and precipitation that sequester or immobilize metal(loid) ions and hence decrease their content in groundwater are described [77]. The mechanism of adsorption is determined by the physicochemical properties of the adsorbent and heavy metals, as well as the operating circumstances (i.e., temperature, pH value, adsorption time, adsorbent amount, and initial content of metal ions) [78]. The geochemical characteristics of sediments strongly govern heavy metals adsorption behavior [79]. Metal mobility in soils is influenced by the pollutants’ structure, their physiochemical characteristics, and the soil’s attributes [74]. While adsorption is feasible for extracting trace heavy metals from wastewater, adsorbents’ affordable and environmentally friendly regeneration is frequently a limiting factor in its adoption [80]. According to [81], the current scientific understanding of water contamination mechanisms is inadequate to properly control the problem in China. Adsorption and desorption are the fundamental mechanisms underlying the interactions of heavy metals with water and sediments [79]. The surface charge of an adsorbent in a solution can be adjusted by altering the pH valve, and the pH is one of the key parameters impacting the adsorption of the metal ions [82]. For instance, rice and other plants absorb more cadmium ions during the winter months because the pH of the soil is lower in the winter months; according to this, irrigating winter fields at important stages of crop development may help improve soil pH and reduce cadmium absorption [83]. Clay minerals significantly influence the bioavailability of heavy metal ions [75]. Numerous factors, including viscosity, porosity, permeability, adsorption processes, solid heterogeneity, and size distribution, might affect ion transport [84]. Only a trace amount of free metal ions remain dissolved in water due to hydrolysis, adsorption, and co-precipitation, whereas a considerable proportion is deposited in the sediment [85]. Adsorption is one of the most convenient ways for tracing heavy metals (<1 mg/L) subsequent co-precipitation due to its simplicity and high performance [80]. As water moves from the soil to the roots via transpiration, organic molecules and ions are transported by diffusion, microbial assistance, and microbial transport [86]. Zn’s high mobility and bioavailability demonstrate that organic bonds can be rapidly reversible, depending on the metal ion [79].

The bioavailability, toxicity, and mobility of toxic metals and metalloids are influenced by speciation [87]. Moreover, it is crucial to highlight that interaction between complexing ligands and the heavy metals directly dependent on their bioavailability and mobility and the sorption-desorption equilibrium between the heavy metal and ligand. Soil characteristics and environmental conditions influence heavy metal bioavailability in soil. Also, soil pH and organic matter impact heavy metal speciation and bioavailability [75]. During bioavailability investigations, the predicted dose should represent the unfolding scenario, and soil particle size has a crucial impact on the exposure routes.

On the other hand, finer particles contain elevated metal(loid)s concentrations because they have higher specific surface states due to more visible mineral surfaces. In many circumstances, the material is not immobilized or segregated becoming a cause of particulate matter, including heavy metals pollution [88]. Particulate matter deposition on soil is a major source of soil pollution with heavy metals, and we must need to address this problem [89]. They also include iron, clay minerals, manganese, organic matter, and aluminum oxides to form fine-sized aggregates [90]. Soil aggregates have a remarkable influence on the distribution and bioavailability of heavy metals. Soil aggregates are composed of various fractions of micro-and macro-aggregates that have formed due to different biogeochemical cycles [75].

Several different mechanisms, including biosorption, intracellular accumulation, precipitation, and redox reactions, have been discovered by researchers to affect the bioavailability of heavy metals in soils [91]. From soil to crop, the bioaccumulation of metal(loid)s differs according to soil-cropping methods [92]. According to several researchers, Pb in mining wastes has a reduced bioavailability. Pb uptake was lowest in samples collected from tailings, smelter slags, and mining soil [44]. Lead minerals may be trapped within other soil mineral grains, such as quartz, at mining and smelting sites, limiting their bioavailability [93].

Nevertheless, absorption by the human body appears to be inferior in mining regions than in smelter sites for a given soil or dust Pb concentration [94]. Among the nine mining and smelting locations in the Hunan region, the soil around this smelter contains high concentrations of lead, zinc, cobalt, copper, and arsenic, all of which have the potential to be highly bioavailable [33]. Additionally, Cd’s bioavailability and/or gastrointestinal bioaccessibility in food and soil should be assessed [46].

### 3.2. Pollution Indices

Pollution indicators are critical for determining the extent of heavy metals soil pollution and the possible environmental hazards [95]. The most popular indexes used for detecting the metal concentrations in the contaminated environments include the geo-accumulation Index (*I-geo*), Contamination Factor (*CF*), Threshold Pollution Index (*PIT*), Pollution Index (*PI*), and Enrichment Factor (*EF*). The existence and intensity of man-made pollutant deposition on the environment are measured using several indices are reported in many studies. The classification criteria of indices are shown in Table 1.

#### 3.2.1. Geo-Accumulation Index (*I-Geo*)

Geo-accumulation index presented by [96] to recognize and describe metal pollution in soils by relating the prevailing concentration levels with pre-industrial levels, *I-geo* is calculated as follows:

(1) $$I-geo=log2\;(\frac{Cn}{1.5Bn})$$

where *Cn* denotes the measured content of metal (*n*) in samples (mg/kg), *Bn* denotes the geochemical background value of metal (*n*) (mg/kg), and the constant 1.5 allows us to define the condition of environmental variability in the content of a given substance and determine minor man-made effects [97]. The *I-geo* has seven grades/classes, ranging from class 0 to 6, as indicated in (Table 1). Table 2 shows the average *I-geo* values and pollution index (*PI*) with heavy metals in soils near non-ferrous smelteries in China and discussed in detail in [41]. Researchers reported using the *I-geo*, which helped us understand soil contamination sources and potential risks.

#### 3.2.2. Pollution Index (*PI*)

The *PI* was determined to indicate the amount of heavy metal contamination [98].

(2) $$PI=\frac{Ci}{Cb}$$

where *Ci* is the content of metal element *i* in topsoil samples and *Cb* represents the target element’s background value.

#### 3.2.3. Enrichment Factor (*EF*)

The enrichment factor defines whether the elemental level is enhanced from natural or anthropogenic origins [99]. *EF* was calculated using the following formula:

(3) $$EF=\left(Ci/Cn\right)s/\left(Bi/BMn\right)b$$

where *Ci* denotes the metal element *i* concentrations in the sample, *Cn* is the elemental reference values for; Generally, elements including Mn, Al, Sr, Fe, and Ti were utilized as references when calculating *EF* values, respectively, and *s* and *b* denote the sample and background value, respectively.

**Table 1:** Classes of single indices of pollution.

Index	Formula	Range of Indices	Soil Conditions	References
Geoaccumulation Index (*I-geo*)	$I-geo=log2\;\left(\frac{Cn}{1.5Bn}\right)$	*I-geo* ≤ 0	Unpolluted	[96]
		0 ≤ *I-geo* < 1	Unpolluted to moderately polluted	
		1 ≤ *I-geo* < 2	Moderately polluted	
		2 ≤ *I-geo* < 3	Moderately to strongly polluted	
		3 ≤ *I-geo* < 4	Strongly polluted	
		4 ≤ *I-geo* < 5	Strongly to extremely polluted	
		*I-geo* > 5	Extremely high polluted	
Pollution Index (*PI*)	$PI=\frac{Ci}{Cb}$	*PI* < 1	Unpolluted, Low level of pollution	[98]
		1 < *PI* ≤ 3	Moderate polluted	
		3 ≤ *PI*	Strong polluted	
Enrichment Factor (*EF*)	$EF=\left(Ci/Cn\right)s/\left(Bi/BMn\right)b$	*EF* < 2	Deficiency to minimal enrichment	[99]
		*EF* = 2–5	Moderate enrichment	
		*EF* = 5–20	Significant enrichment	
		*EF* = 20–40	Very high enrichment	
		*EF* > 40	Extremely high enrichment	
Contamination Factor (*CF*)	CF=Cs/CRe fS	*CF* < 1	Low contamination factor	[100]
		1 < *CF* ≤ 3	Moderately contaminated factor	
		3 ≤ *CF* ≤ 6	Considerably contaminated factor	
		6 ≤ *CF*	Very high contaminated factor	
Threshold Pollution Index (*PIT*)	PIT=Ci/CTL	*PIT* < 1	Unpolluted	[101]
		1 < *PIT* ≤ 2	Low polluted	
		2 ≤ *PIT* ≤ 3	Moderate polluted	
		3 ≤ *PIT* ≤ 5	Strong polluted	
		5 ≤ *PIT*	Very strong polluted	

#### 3.2.4. Contamination Factor (*CF*)

(4) CF=Cs/CRe fS

where *Cs* is for total soil element content (mg kg^−1^), while *CRefS* stands for reference content in pristine soils (mg kg^−1^), “world-wide average” [100].

#### 3.2.5. Threshold Pollution Index (*PIT*)

(5) PIT=Ci/CTL

where *Ci* is the content of the heavy metal *i* determined, *CTL* is the tabulated minimum content determined heavy metal [101].

These indices, including geo-accumulation index (*I-geo*), pollution index (*PI*), enrichment factor (*EF*), contamination factor (*CF*), and threshold pollution index (*PIT*), have been invented by researchers to estimate metal enrichment in water and surface soils. Even though pollution indices are beneficial for identifying and classifying metal contamination, they do not give insights into the potential existence of biological consequences linked with contaminated sediments. Indeed, such indexes were not intended for that purpose. Future research on environmental assessments using pollution indices around non-ferrous smelting facilities and tailing ponds containing metal ore post-flotation waste is needed as a preliminary step.

### 3.3. Heavy Metal Contamination in Smelter Impacted Regions

Due to fast population growth and depletion of natural resources, heavy metals contamination has turned into a considerable worldwide environmental issue, impacting a global scale. There is indisputable proof that the mining and smelting sectors significantly impact environmental destruction [102]. Numerous soil pollution studies have indicated that smelter-polluted areas in various countries have extremely high amounts of toxic heavy metals like Cu, Pb, Ni, Zn, Cd, As, and Hg [103]. But the largest concentrations of meta(loid)s were typically found in the soil’s surface layers [48]. According to previously published results, environmental contamination and its associated impacts are widespread in south-central and south-western China and along the coasts of Zhejiang, north Henan, Liaoning, and Fujian, as a result of lead/zinc mining and smelting processes [8]. Table 3 illustrates the distribution of heavy metal contents at smelting areas in China.

Under current global environmental regulations for safety and control norms, Arsenic is a challenge for the mineral processing sector [104]. Arsenic pollution may be widespread at mining and industrial sites, necessitating a hazard assessment that evaluates the potential mobilization of As in soils [105]. Arsenic levels in topsoil were elevated in the vicinity of the Pb and Zn smelter manufacturers [106]. The average arsenic content was 35.1 mg As kg^−1^ (range: 3.56–205 mg As kg^−1^), exceeding 205.1 mg As kg^−1^ due to contamination by the arsenic residue of the alkaline incinerator [107]. According to China’s soil pollution standard, 40% of the surface soil samples collected at those locations had levels of mercury that are categorized as highly polluted (>1.50 mg kg^−1^), the topsoil appeared to be polluted by Hg deposited from surrounding point sources [108]. Anthropogenic Hg accumulation in topsoil (0–15 cm) is 346–543 kg, and using a 20-year operation time interval; the projected annual Hg deposition is 17.3–27.2 kg (mean of 22.3 kg) [109]. Chinese scientists found that the soil Hg content in artisanal (Zn) smelting regions of Guizhou Province was as elevated as 0.86 mg kg^−1^ [108,110]. Cadmium is a highly active heavy metal in the water-soil-plant system [46]. The root tips of plants can turn yellow and brown due to higher cadmium accumulation, leading to plant mortality. According to [111], studies suggested that the paddy soils were primarily polluted with Cd and antimony (Sb). Still, the quantities of heavy metals in rice grains and their potential health effects on residents were unknown. Hence, it is essential to recognize the heavy metals origins and concentrations throughout the locations. Cd deposition flux as high as 500 mg/m^2^/year in Europe, despite a noticeable reduction in Cd atmospheric deposition in Europe during the 1970s–1980s [23]. Yuguang stack’s atmospheric deposition polluted the farm field near the Yuguang; no pollution from wastewater or solid waste was found [112]. Significant Pb, Cd, and As deposition fluxes around the Yuguang smelter in Jiyuan, Henan Province [23].

Antimony is a non-essential element for plants. It is quickly absorbed by roots in a dissolved soil solution, making it a significant plant pollutant in mining and industrial areas [107]. Radish plants sampled in the Lenshuijiang City, Hunan Province, had significant Sb concentrations; the leaves contained high Sb contents, strongly associated with soil Sb concentration [107]. According to the [113], the following were the ranges of each element (in mg kg^−1^) in polluted and control areas: Ag, 0.63–3.71 (0.28–0.66); Bi, 1.94– 21.40 (0.41–1.84); Co, 3–30 (11–28); Cr, 47–199 (70–115); Ge, 1.28–2.27 (1.38–1.67); In, 0.440–4.220 (0.037–0.155); Ni, 7–58 (20–43); Sb, 5.12–19.60 (0.98–3.92); Sn, 3.7–26.3 (0.5–15.1); Tl, 0.48–1.58 (0.47–0.76), respectively. As shown in Table 3, the highest heavy metals concentrations (in mg kg^−1^) indicated wide variability between various smelting sites, and the ranges for Ag, Hg, Bi, Co, Ni, Cd, Cr, As, Mn, Cu, Sb, Zn, and Pb were 3.71, 15, 21.4, 30, 45.6, 131, 199, 205, 468.70, 716, 5045, 31625, and 37770, respectively. Traces of metal/metalloid such as Ag, Bi, In, Sb, Sn, and Tl are substantially higher in the soils surrounding Zhuzhou smelter than in other Zn and Pb smelters in northern France and Macedonia, although it’s quite comparable to a Pb–Zn mining and smelting area in Kosovo [113]. Cd and Cu are the two most significant metal pollutants, and their concentrations in most soil samples were higher than the guideline levels of China [28].

**Table 2:** Average *I-geo* values and pollution index (*PI*) with heavy metals in soils around non-ferrous smelteries in China, by province. Reprinted from [41], Copyright (2021), with permission from Elsevier.

Province	*n*	*SN*	*I-Geo*				*PI*				
			Cd	Cu	Pb	Zn	Cd	Cu	Pb	Zn	NIPI
Anhui	6	1	-	1.45	−0.27	−0.76	-	0.84	0.11	0.22	0.65
Chongqing	8	1	7.72	-	7.43	-	128	0.00	28.4	-	98.0
Fujian	25	1	-	1.30	4.30	2.67	-	0.84	4.06	3.30	3.46
Gansu	24	2	7.24	−0.49	5.12	4.37	87.7	0.26	3.26	8.53	64.5
Guangdong	17	1	5.04	-	1.97	-	9.23	0.00	0.70	-	6.94
Guangxi	104	3	7.22	2.90	6.43	1.33	206	3.12	10.3	1.14	151
Guizhou	179	6	1.73	1.03	5.99	0.90	10.9	0.98	11.2	1.11	8.99
Hebei	9	1	-	-	3.32	-	-	-	1.07	-	1.07
Henan	131	3	4.79	−0.01	2.51	0.09	10.3	0.29	0.56	0.38	7.54
Hubei	11	2	7.41	4.56	3.56	2.27	147	10.8	1.57	2.42	108
Hunan	47	3	5.89	2.18	3.41	2.97	37.3	1.86	1.57	4.44	27.6
Jiangsu	38	1	-	-	4.26	-	-	-	2.51	-	2.51
Jiangxi	12	1	3.02	3.46	1.37	−0.32	4.37	5.05	0.42	0.33	4.00
Liaoning	94	2	7.39	4.95	4.53	4.40	90.33	9.16	2.47	8.04	66.8
Shaanxi	305	12	6.34	0.53	2.96	5.04	38.00	0.46	0.83	13.7	28.5
Sichuan	46	1	-	-	5.09	-	-	-	5.26	-	5.26
Tibet	17	1	-	-	−0.02	-	-	-	0.14	-	0.14
Yunnan	436	3	4.54	1.65	2.89	3.48	25.3	2.18	1.51	6.00	19.0
Zhejiang	284	4	4.32	3.51	2.53	3.13	7.00	3.01	0.68	3.70	5.57
China	1793	49	5.59	2.08	3.54	2.27	61.65	2.59	4.04	4.10	32.0

*n* donates datum number collected from the literatures; *SN* donates smelter number of provinces; - denotes the data were unavailable in the public reference.

**Table 3:** Descriptions of studies dedicated to soils around smelteries in China. * This data is “not available”.

City	Locality	Smelting Operation	Contaminants (Maximum Concentrations in mg/kg)	Spatial Distribution	Soil Profiles	Mineralogy	Extractions	Isotopes	Bioavailability	Digestion	Measurement Method	Reference
Hunan	Zhuzhou (Hunan)	Pb/Zn smelter	Zn (3349), Pb (1197), Cu (157), As (93), Cd (41.1), Hg (2.89)	* (Topsoils)	*					HNO_3_-HCl-H_2_O_2_	AAS	[33]
Hunan	Zhuzhou (Hunan)	Pb/Zn smelter	Ag (3.71), Bi (21.4), Co (30), Cr (199) and other 6 elements	* (Topsoils)	*					HF HNO_3_	ICP MS	[113]
Hunan	Xikuangshan (Hunan)	Sb smelter	Sb (5045), As (205)		* (Topsoils)		*		* (Radish)	HCl-HNO_3_	ICP-AES	[107]
Hunan	Zhuzhou Hunan	Zn/Pb Smelter	Hg (1.54)	*	*						ZAAS-HFM	[108]
Henan	Yuguang Henan	Pb smelter	Cd (2.10), Cu (31.8), Ni (45.6), Pb (184), Zn (99.2)		* (Topsoils)					H_2_O_2_ HF HClO_4_ HNO_3_	AAS	[53]
Henan	Jiyuan Henan	Pb smelter	Pb (114 ± 2.17) Cd (1.19 ± 0.049)						(Wheat)	HNO_3_-H_2_O_2_	AAS	[112]
Zhejiang	Hangzhou (Zhejiang)	Cu smelter	Zn (11,840), Cu (716), Cd (8.67)		*		*			HCl/HNO_3_	FAAS	[114]
Zhejiang	Fuyang (Zhejiang)	Cu smelter (secondary)	Hg (15)	* (Topsoils)	*					HNO_3_-H_2_O_2_	AFS	[109]
Zhejiang	Zhujiawu (Zhejiang)	Cu/Zn smelter	Zn (3219), Cu (658)	* (Topsoils, transect)					* (Microbes)	HF-HClO_4_-HNO_3_	FAAS	[115]
Jiangxi	Guixi Jiangxi	Lengshui Pb-Zn mining & copper smelter	Mining Cd (0.84 ± 0.63) Smelting Cd (1.08 ± 0.36)		*				(Vegetables)	HNO_3_, HF, and HClO_4_	ICP-MS	[46]
Jiangxi	Dexing Jiangxi	Pb-Zn, Au, Cu mines	As (33.99), Cd (1.22),Cr (70.28), Cu (138.42), Mn (468.70), Ni (32.24), Pb (125.32), Zn (171.48)	* (Topsoils)	*					HNO_3_-HClO_4_-HF	ICP-AES	[28]
Jiangxi	Guixi Jiangxi	Cu smelter	Cu (35)		* (Topsoils)				(Rice)	(HNO_3_), (HF), (HClO4)	ICP	[17]
Guizhou	Magu (Guizhou)	Zn smelter	Pb (37,770), Zn (31,625), Cd (131)		* (Topsoils)	*	*	* (Pb,S)		HF-HClO_4_	AAS	[116]
Hubei	Daye Hubei	Cu smelting	Cd (4.87), Cu (195.26), Pb (92.65), As (35.84)						* (Crop)	HClO_4_, HNO_3_ and HF, HClO_4_ and HNO_3_, and HNO_3_ and H_2_O_2_	ICP-MS	[117]
Guangxi	Nanning (Guangxi)	Pb/Sb smelter	Pb (992), Zn (597), Cu (39), Cd (22) (geometric means)	* (Topsoils)					* (Vegetables)	Soil HNO_3_/HCl = 1:3	ICP-MS	[118]
China	19 major provinces	49 Non-ferrous smelteries	Cd (19.8), Cu (265), Pb (1536), Zn (1371)	*						HCl_4_-H_2_SO_4_, HCl-HNO_3_, HCl-HClO_4_-HNO_3_, HCl-HClO_4_-HF-HNO_3_, HCl-HClO-HNO_3_, HF-HNO_3_, HCl-HF-HNO_3_, HClO_4_-HF-HNO_3_, HCl-HF-HNO_3_-H_2_O_2_, HCl-HNO_3_-H_2_O_2_, HClO_4_-HNO_3_,	(AAS), (ICP-AES), (ICP-MS), (ICP-OES)	[41]

Lead concentrations (C_Pb_) of more than 100 mg kg^−1^ have been detected in topsoil throughout a 106 km^2^ area surrounding the two smelters [119]. A significant content of heavy metals in soil has resulted from long-term mining and smelting activities, including Pb, due to Cu and Pb-Zn mines [120]. Pb smelting operations in the province of Henan have already had a significant environmental impact [121]. The findings reveal that atmospheric deposition from the lead smelter significantly increases the availability of Pb and Cd in the soil much further than normal levels and the availability of Ni, Cu, and Zn [32]. Previous studies revealed Cd and Pb as the principal contaminating elements in soils around lead smelters in Henan Province [53]. The Pb concentration is normally substantially greater than Cd in smelter-affected soils [53]. Numerous researchers have found that the lead and zinc mines and smelting operations significantly contribute to soil pollution [102]. For example, 1793 surface soil samples taken from 49 non-ferrous smelting locations in several regions revealed mean Cd concentrations of 19.8 mg/kg; Cu concentrations of 265 mg/kg; Pb concentrations of 1536 mg/kg; and Zn concentrations of 1371 mg/kg [41]. According to the long list of pollutants discovered, Pb is the most common pollutant detected in urban soils and is frequently present at alarmingly high levels. It can be challenging to determine the exact source of high soil-lead concentrations in any specific site.

Non-ferrous metal mines have severely contaminated agricultural soils in the Jishui River Valley [28]. Additionally, geochemical speciation data of sedimentary heavy metals revealed that Cd, Ni, Co, and Pb greater environmental risk is due to their higher availability in the exchangeable fraction, which negatively influences aquatic biota [122]. Water is an essential resource to sustain life on this planet [123]. In 2012, 31% of water quality monitoring locations along seven major Chinese rivers had weak water quality (grade IV). Amongst the degraded monitoring locations, fifty percent of the monitoring stations along the Huaihe River were seriously polluted. The Huaihe River’s water quality has deteriorated in recent decades, and substantial regions of the province do not fulfill national surface water quality criteria [124]. In addition, the river water around the smelter was heavily polluted by heavy metals. For example, the discharge of waste containing high contents of heavy metals from Pb smelting operations into local rivers has resulted in severe water and sediment pollution in Jiyuan [23]. Also, the exposed area’s well water may have become polluted with Cd and As due to smelting activities [117]. Stormwater sweeps all sorts of pollutants from various land practices to the river [124], and irrigation of farmland with heavy metal polluted river water posed a serious threat to soil health [125]. Irrigative sewage transports heavy metals to the soil, where they are fixed in a variety of ways. It causes a constant accumulation of heavy metals (Pb, Cd, Cr, Hg, etc.) in the soil year after year. Commercial wastewater release is a significant Pb contamination source in the Yangtze River Delta and Pearl River Delta [18]. Due to frequent water pollution incidents, the Huaihe River, China’s seventh-largest river, poses a severe threat to its national security and socio-economic development [124]. The Huaihe River basin is China’s primary grain-producing region [124]. Little water resources, population increase, and climate change have caused freshwater problems in numerous nations. While climate change and industrialization further threaten the fragile situation in this region. So the government, academics, industry, and the general people are all needed to help prevent and control soil pollution.

### 3.4. Distribution and Source of Heavy Metals

**Figure 1:**
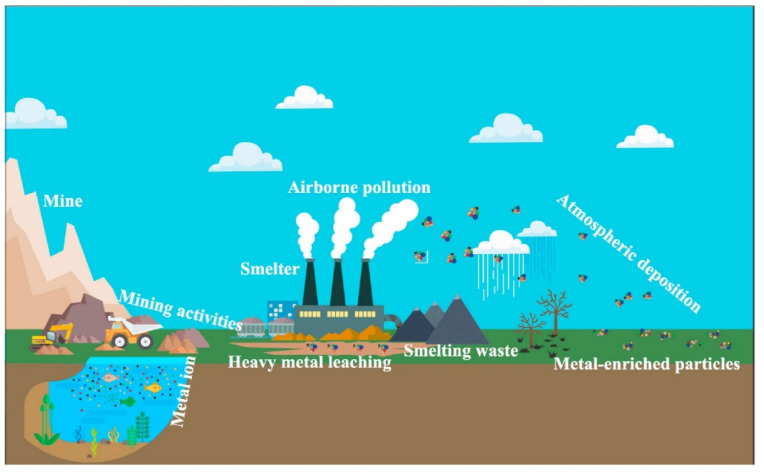
Schematic illustrating pollution sources of heavy metals in smelter polluted soils. Reprinted from [103], Copyright (2021), with permission from Elsevier.

The spatial distribution of heavy metal contents is a helpful tool to identify potential origins of enrichment and hotspots with high heavy metal contents in the environment [126]. The elevated contents of metal oxides, OM, and clay minerals in soil particles play a significant function in the mobility and bioavailability of soil heavy metals [127]. Consequently, atmospheric deposition is the major mechanism by which heavy metals from stacks reach the soil near the smelters [23]. Gas and dust created by energy, transportation, metallurgy, and construction materials are the principal sources of heavy metals in the atmosphere. The geological history level of heavy metals in China is low. As shown in Figure 1, it’s potential to conclude that heavy metals concentrations in soil can be generated by a wide range of characteristics, including fertilization and agrochemical application; sewage irrigation; atmospheric deposition; mining; the sludge application and smelting procedures for metallic ores; combustion of fossil fuels; industrial wastes; and refinishing. Heavy metals contaminate the environment through nonferrous metal mining and smelting, solid waste weathering and leaching, the release of contaminated water, or particles from smelters’ stacks decomposing in the atmosphere [112].

It was estimated that more than 9.45 × 10^5^ tonnes of wastewater were released into the surrounding environment, 6.72 × 10^5^ tonnes of waste from mining and smelting, and 1.50 × 10^5^ tonnes of arsenic-alkali were released into the surrounding environment in 2007 [128]. Mercury emissions from nonferrous metals smelters accounted for about 38% of the anthropogenic atmospheric mercury in China [129]. China has an abundance of (Sb) mineral resources. The Xiquangshan Sb mine is one of the world’s largest Sb mines near Lenshuijiang City, Hunan Province. It has long been regarded as the “World Antimony Capital.” [107]. China is a significant contributor to the global man-made Sb emissions due to its high Sb production and overall atmospheric Sb emissions from coal-burning [130].

In some cases, heavy metals have been linked to soil parent materials; in other cases, soil parent materials have been linked to a variety of heavy metal species. The amount of selenium in the soil varies by geographic region natural and artificial sources of selenium in soil; soil generated by carbon-rich sedimentary rocks contains more selenium, while soil formed by magmatic rocks contains less. In 2018, China produced 1.98 × 10^7^ tons of Pb, Cu, and Zn from smelting, accounting for 50.5 percent of global production [41]. In Guizhou, there were six smelteries, all Zn-smelteries [41]. In China, Jiyuan City is significant for Zn, Pb, and Coal Production [53].

According to the previous study, a high metal concentration in the smelter-derived particles (e.g., particles from a Pb smelter contain 53.6–62.8% Pb, 1.2–1.8% Cd, 3.8–5.5% Zn, 0.1–0.6% As, 0.07–0.26% Cu, particles from a Zn smelter contain 45.76% Zn, 15.66% Pb, 0.57% Cu, 0.57% As, 0.014% [33]. In China, the smelting of non-ferrous metals has become the largest source of contamination, with enormous extents of Cd, Cu, Pb, and Zn released into the soil [41]. Cd is an extremely toxic metal that imposes serious hazards to the environmental [130]. That occurs naturally and is generated mainly through industrial sources, including mining and metal smelting [46]. In 2010, nine provinces released above 100 tons of y^−1^ Cd, most in central, south, and southwest China [131]. The Guixi Copper Smelter in Jiangxi Province is China’s largest flash Cu smelter, the main source of copper contamination in the adjacent environment [17].

China has become one of the countries with more copper smelting capacity, processing about 40% of copper concentrates worldwide [104]. Much of this contamination has been attributed to Pb/Zn smelters. Hunan Province, in central China, is one of the country’s leading producers of non-ferrous metals [33]. Daye City in Hubei Province is well-known for its vast mineral resources and advanced smelting processes. In recent years, Henan Province has generated approximately 1.5 million tons of lead per year, while Jiyuan City was producing 0.9 million tons [23]. Smelter emissions are estimated to account for 40–73 percent of anthropogenic productions of heavy metals into the environment [103].

### 3.5. Health Risk Assessment

To define and measure the toxicity features of heavy metal emissions and their harmful impacts on the environment. Humans and the environment can be affected by pollutants emitted by mining activities. Still, due to human activity, heavy metals pollute the air, water, soil, and plants occasionally and can even impact human health via the food web [132]. The soil hazard elements are difficult to decompose. They can pass through food chains and water supply systems to plants and humans, posing a direct or indirect threat to food security and public health [133]. Humans and animals can ingest heavy metals in the environment in various ways, such as drinking water, food, dermal contact, and inhalation [125]. Heavy metal contamination has generated intense environmental and health-related issues around the globe [1]. Heavy metal contamination nearby smelting sites have high Pb and Cd levels in crop grains, posing a health risk to residents [112]. While Cu is a necessary trace element for life, excessive levels can endanger human health and negatively affect animal and plant growth [17]. The main path of human exposure to soil contamination is via a soil-to-plant transfer of heavy metals. To produce high-quality food and maintain ecological balance, agricultural land must be conserved [134].

Previous research reported that 38% of kids and 5% of adults in the smelting region surpassed the health standard for Cd in hair (<0.5 mg kg^−1^ for children and <0.6 mg kg^−1^ for adults) stated by the Trace Element Research Council of China [46]. In China and Japan, lifetime exposure to low Cd contamination causes renal dysfunction in inhabitants living near cadmium-contaminated soils [118]. Children in the Daye Copper Smelter (DCS) region were exposed to Cd, As, and Pb exposure levels up to 30.25 times higher than the acceptable level of 1, and most hazards were through ingestion of soil, drinking water, crops, and fish, according to [117]. Also, the USEPA has recognized arsenic as the primary carcinogen that causes cancer in humans when ingested [77]. Certain Sb compounds are believed to be dangerous to human health—even cancerous [107]. The previous result showed that inorganic As in food causes 9129 to 119,176 extra bladder cancer cases annually, 11,844 to 121,442 additional lung cancer cases, and 10,729 to 110,015 additional skin cancer cases worldwide [92]. Excessive Zn may cause skin irritations, vomiting, and stomach cramps, whereas excessive Ni may result in lung and kidney cancer [135].

Dermal absorption is the primary route of exposure for As, Cd, Cr, Cu, Ni, and Hg, whereas ingestion is the primary route of exposure for Pb and Zn. For instance, on the Daxin manganese mine site, the average daily intake of Cr by adult men via the dermal absorption pathway is 3.2 × 10^−1^ mg/kg-day [123]. Children are especially susceptible to Pb exposure caused by frequent hand-to-mouth action, faster absorption rates, and a developing Central nervous system [136]. Due to the increase of the non-linear cumulative risk of increasing blood lead levels (BLLs), children’s BLLs have been split into five levels of reducing size intervals: 0–30.00 μg/L, 30.01–50.00 μg/L, 50.01–60.00 μg/L, 60.01–65.00 μg/L, and 65.01–70.00 μg/L [137]. There is limited evidence that local children have elevated BLLs, which may be related to the harmful impact of Pb smelting on local inhabitants [36]. Blood samples from 1008 of 3108 children (32 percent) residing nearby lead smelters in Jiyuan City, Henan Province, revealed lead amounts greater than 250 μg/L [138]. Heavy metal soil pollution and higher BLLs have been observed in Jiyuan [23]. Additionally, Pb was discovered in people living adjacent to the smelters [53]. Even in north China, Pb and Cd pose a health risk to surrounding residents [32].

The study’s findings established that discharges from smelting and transportation activities were the primary contributors to non-carcinogenic hazards [139]. For non-carcinogenic risk, 1 is the acceptable level [9]. According to the USEPA’s recommendation, when the levels of Hazard Quotient (HQ) and Hazard Index (HI) are more significant than 1, there may be a detrimental effect on human health [111]. According to [8] investigation, the worst-case scenarios were assumed by using children’s non-carcinogenic hazard values. That indicates that the non-carcinogenic hazard values associated with mining regions in southern provinces (e.g., Hunan and Guizhou) are higher than those in other areas. Observed that the potential non-carcinogenic hazard for kids was 1.2 times more than the hazard for adults, indicating much more significant harmful impacts on children [46]. Besides that, the previous studies find that the hazard quotient (HQ) maximum values at Pb/Zn smelting locations in Guangdong, Sichuan, Guizhou, Guangxi, Jiangsu, Hunan, Liaoning, and Zhejiang were greater than 10, while the mean and median HQs in Sichuan, Fujian, Guangxi, and Zhejiang were between 1 and 10, indicating that Pb-contaminated surface soils in these regions may pose risks to neighborhood kids [50]. Notably, Hunan Province has higher BLLs than other areas, owing to its abundant nonferrous metal and nonmetallic mineral reserves and severe soil pollution [18]. Provinces with high hazards (50.01–60.00 g/L) are primarily situated on the coast (Zhejiang, Shandong, and Jiangsu) and in central (Henan and Hunan) and western areas (Qinghai, Gansu, and Shaanxi) [137].

**Figure 2:**
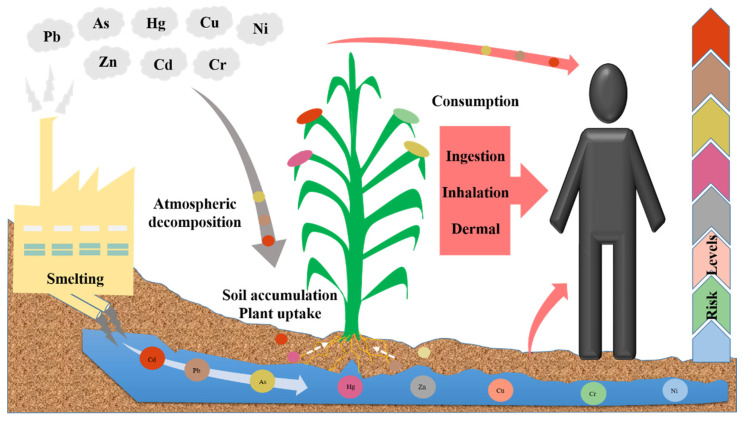
Release of heavy metals and their dominant exposure and possible uptake routes to humans.

Therefore, several common metal pollutants, including Cu, Ni, and Zn, are vital micronutrients for human, plant, animal, and microorganism health. Exposure to heavy metals relies on the metal’s characteristics. Heavy metals can accumulate inside the human body and cause serious health issues even at low contents. Identifying soil pollutants and their origins is vital to research because of their close link to human health. In the context of heavy metal pollution from smelting operations, it is essential to carefully examine the potential environmental and human health hazards. (Figure 2), provides a brief overview of the health and environmental hazards generated by heavy metals pollution. In this area, the health risk of inhabitants caused by exposure to soil heavy metals must be of significant consideration. As a safety measurement, it is suggested that new factories be located further away from residential areas to minimize the influence of contamination on human health. Future research in this area, which focuses more on remediation technologies, could bring more cost-effective and long-lasting methods of treating soil and water.

### 3.6. Metallurgical Processes

Waste from industries, pharmaceutical businesses, hospitals, e-waste, and other non-segregated waste that ends up in landfills contains various hazardous elements. The inappropriate disposal of waste products negatively affects the environment and is a significant threat to public health. Nevertheless, landfill failures can release large volumes of harmful heavy metals into the adjacent soil and groundwater. Industrial metallurgical processes are commonly used to recover metals from metal ores. Several metallurgical processes for extracting metals from waste, such as hydrometallurgy, pyrometallurgy, electrometallurgical, and biometallurgical processes, have been developed. However, pyrometallurgy and hydrometallurgy are the most commonly employed technologies in industrial applications to recover metals from waste.

The pyrometallurgy process is a chemical method for extracting and purifying metals, and it passes through roasting, smelting, and refining processes [140]. This approach has a number of disadvantages, including a high energy requirement and the need for dust collection/gas cleaning equipment.

#### Hydrometallurgy Processes

**Figure 3:**
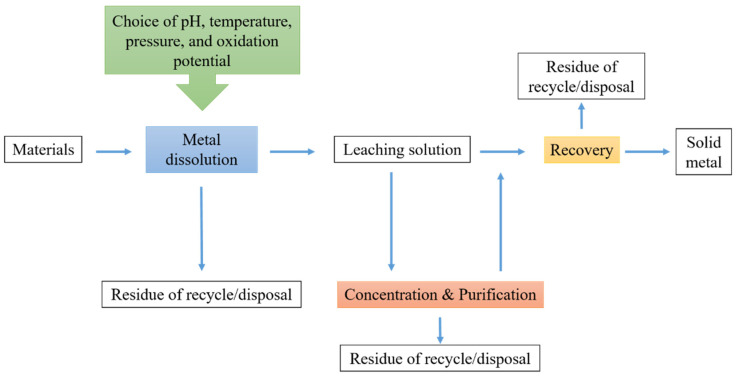
Basic unit process of hydrometallurgical metal extraction (modified from [145]).

Hydrometallurgy can be described as a metal recovery process that uses aqueous media and combines water, oxygen, and other chemical reagents with or without a pressure environment to extract metals from ores and waste products [141]. Hydrometallurgical processes are more selective and thus more efficient while also reducing pollutants. During the last five years, China’s research on recovering vanadium from vanadium slags has increased dramatically [142]. Waste can be segregated and treated to make new products, and waste is not a waste until it is wasted. For instance, Fuyang city in Zhejiang province, China, is one of the largest national reuse centers for secondary Cu industries, recycling waste copper [109]. However, most current investigations have focused on using different types of microbial strains to bioleach heavy metals from industrial sludge and convert them to a solubilized state [141]. Metals are dissolved in an alkaline or acid medium in hydrometallurgical processes. Upscaling and control processes are more flexible with hydrometallurgical operations [143]. Furthermore, mechanical–physical, dismantling, pyrometallurgy, or hydrometallurgy can create and discharge heavy metals (e.g., Cu, Pb, Cd, Cr) and persistent organic pollutants (POPs) during recycling and deep extraction for precious resources (e.g., polychlorinated biphenyls, polycyclic aromatic hydrocarbons, polybrominated diphenyl ethers, and polychlorinated dibenzo-p-dioxins and dibenzofurans) [144]. Hydrometallurgy can be divided into leaching, concentration and purification, and metal recovery [145] (Figure 3).

Leaching is the process of immersing a precious metal in aqueous solutions containing a lixiviant. The oxidation potential, temperature, and pH of the solution are critical factors in the leaching process. They are frequently changed to optimize the dissolving of the desired metal component into the aqueous phase. In-situ preaching, heap leaching, and vat leaching are the three essential leaching processes. Bioleaching may be a viable alternative treatment approach for solid waste materials with low concentrations of valuable metals or otherwise difficult to manage or treat [146]. Bioleaching of e-waste is only conducted at the lab level, and future pilot-scale research is required to establish it as an industrial metal leaching approach related to the current hydrometallurgical processes in developing countries [147]. It is divided into different leaching processes based on the leaching solution, such as acid, alkaline, thiosulfate, thiourea, halide, and cyanide. The reduction of total heavy metals content in electroplating sludge is not the only standard for assessing the efficacy of acid leaching [148]. These processes use ecologically harmful substances to solubilize metals [149]. Acid leaching is used to separate metals into two groups: less valuable metals (Cr and Fe) and valuable metals (Cu, Ni, and Zn) [150].

Concentration and purification steps typically involve solvent extraction, adsorption on activated carbon, and ion exchange. The leach liquor must normally generally concentrate on the metal ions to be extracted. Solvent extractions extract dissolved metals, thus causing it tough to selectively isolate useful metals from electroplating sludge for recovery [150]. The key advantages of solvent extraction are the selectivity and elevated purity of targeted metals and the capacity to treat large amounts of material at once [141]. And it’s also utilized for the extraction of useful metals. By modifying the properties of resins, particularly their functional groups, ion exchange can be successfully exploited for selective separation and recovery of metal ions [141]. To exchange cations or anions with the solution, chelating agents, natural zeolite, activated carbon, resins, and liquid organics impregnated with chelating agents are all utilized.

Metal recovery is a term that refers to the process of extracting metal from solution in solid form, either chemically or electrochemically. Precipitation, crystallization, solvent extraction, ion exchange, electrowinning, and other processes are used to recover metal from the leach solution. Electrowinning is a method of producing metal. Before electrowinning, the iron is precipitated. After purification, the solution is acceptable for zinc recovery using electrolysis. Electrowinning is then used to recover zinc from the purified solution [151]. Electrowinning is a single technique for heavy metal removal that yields no extra waste and may be directly used to generate high-quality metal deposition from solutions without the need for extra chemicals [152].

Numerous metallurgical processes are being used to extract metals from e-waste, but hydrometallurgy is a much more eco-friendly and cost-effective process for recovering metals from industrial sludge. Compared to pyrometallurgy, just a fraction of the gases are discharged into the environment throughout the procedure. Although several remediation approaches have been developed to treat e-waste; however, several issues still need to be addressed before these methods can be implemented. Scientists and government officials must develop a management strategy for e-waste production to reduce the potential consequences of waste.

## 4. Remediation Approaches

Heavy metals are widely found in Chinese abandoned industrial sites. Heavy metals in soil can threaten human health by poisoning groundwater and affecting the soil. Environmentally friendly and cost-effective remediation of polluted areas is a big challenge [153]. Hence, remediating smelter sites has been a significant problem for the government and academia [103]. According to the European Environmental Agency, the European Union 32 member countries observed 250 000 contaminated sites, most of which were polluted with heavy metals and mineral oil [154]. The Ministry of Land and Resources released the consequences of a countrywide soil contamination investigation, finding that 16% of the Chinese soil samples surveyed, comprising 6.3 million km^2^, were contaminated [155]. Since over 2 million hectares of soil in southern China have been polluted by mining and smelting activities, it is critical to apply cost-effective phytoremediation to repair such heavy metal-affected soil [156]. As shown in a 2014 Chinese “National General Survey on Soil Contamination,” 34.9 percent of abandoned industrial sites and 33.4 percent of mining sites are polluted with heavy metals, predominantly lead (Pb) and cadmium (Cd) [157]. Remediating polluted sites protects both human and environmental health. It has evolved from a specialized field to a thriving industry, with a multi-billion dollar market in Western countries and a rising market in China. Historically, clean-up was mainly focused on reducing contamination risk [158].

**Figure 4:**
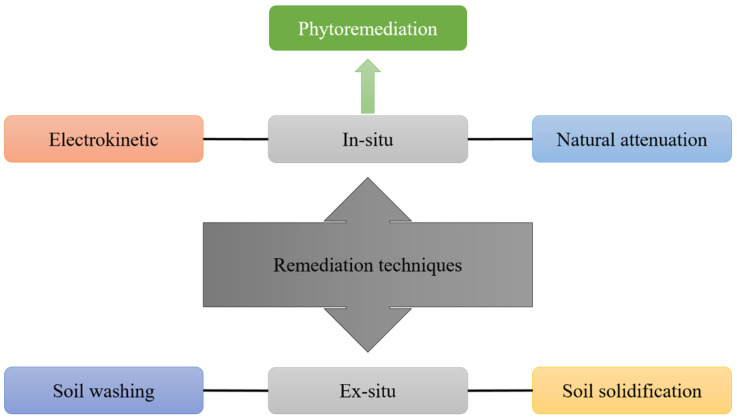
Classification of basic remediation approaches.

Numerous remediation procedures have been established to treat soil, leachate, wastewater, and groundwater polluted by numerous contaminants, including in situ and ex-situ approaches [159], such as surface capping, soil flushing, electrokinetic extraction, solidification, vitrification, and phytoremediation, have been developed to contain, clean up, or recover heavy metal polluted soils [160]. Physical, chemical, and biological approaches are utilized to clean up heavy metal polluted soils [161]. Volatilization by air venting, leaching with a surfactant, vitrification (in which pollutants are solidified with an electric current), and isolation and containment via physical barriers are all in situ procedures. Excavation followed by thermal treatment, chemical extraction, and/or solidification (encapsulation) before landfill disposal are ex-situ procedures [86]. Various treatment methods, including stabilization/solidification, thermal desorption, in situ vitrification, soil flushing, and soil washing, may be utilized to remediate Hg polluted soil [162]. As shown in (Figure 4), we studied various remediation procedures for heavy metal contaminated soils. These remediation procedures will be explained below in considerable detail.

### 4.1. Soil Solidification/Stabilization Technology

In situ, solidification/stabilization processes are preferable due to reduced labor and energy costs. Site factors such as bedrock, huge boulders, clays, and greasy areas may create mixing issues. In situ procedures are best suited for shallow contamination because traditional apparatus such as draglines, backhoes, clamshell buckets, and vertical auger mixers are employed. Vitrification is a thermally induced solidification/stabilization process. Additionally, mixed wastes can be processed in this manner [70]. In complicated hydrogeologic environments, in situ vitrification works well [159].

### 4.2. Soil Washing/Extraction Technology

Soil washing/extraction has recently received much attention as a physicochemical remediation procedure because of its high removal efficacy, ease of use, and cost-effectiveness [163,164]. Ex-situ Soil Washing is often used to treat polluted soils by separating the most polluted fraction of the soil for disposal in a batch system with a specific solid/liquid ratio [165]. However, it removes contaminants from soil by utilizing physical forces (e.g., those caused by propellers/impellers) and liquid solvents (chemical extraction). Soil washing is a promising toxin-contaminated soil remediation technique (removal efficiency: 66–99 percent) [163]. Moreover, on-site or off-site treatment of contaminated soil in large quantities without regard to soil heterogeneity is possible with this method, as is the treatment of mixed pollution (i.e., HOCs and heavy metals at the same time) [165]. Different types of man-made and natural solvents have been studied for different polluted soils in terms of soil washing solvents. Heavy metals from mining soils were washed with four synthetic solvents, including HCl, H_2_SO_4_, HNO_3_, and Na_2_EDTA, according to [166]. However, there have been few investigations on on-site soil washing in paddy fields, although on-site soil washing could be appropriate for paddy fields, which typically feature an impermeable layer that holds the wash solution in the surface layer [167].

### 4.3. Electrokinetic Remediation Technology

Electrokinetic (EK) remediation has been acknowledged as one of the most promising approaches for eliminating various contaminants from fine-grained soil with low permeability since the late 1980s [168]. In China, electrokinetic remediation is growing rapidly [169]. The electrokinetic technique is ineffective at lowering the pH of soils with a high capacity for acid/base buffering [170]. Ref. [171] conventional electrokinetic remediation techniques; 57 percent of the initial Cr and 49 percent of the initial Cd were removed. Electrokinetic soil remediation has removed some heavy metals and organic compounds [172]. It involves transmitting a low-voltage current through polluted soil between a cathode and anode [70]. As a result, using a direct electric current may alter the soil pH, zeta potential, electrolyte content and change the efficacy of electrochemical remediation [171]. Each method of remediation for soil now has some disadvantages, such as high costs (thermal treatments), soil texture perturbation (thermal treatments), low efficiency (pump and treat), long treatment times required (biodegradation processes), or selectivity toward the target pollutant (selective degradation) (volatile organic compounds for venting, hydrophilic organic compounds for pump and treat) [153].

### 4.4. Natural Attenuation

Natural attenuation (NA) is an in situ remedy strategy that employs natural procedures to prevent the spread of pollution from chemical spills [159]. It decreases pollutants through natural methods such as advection, dispersion, sorption, and biotic and abiotic reactions without active intervention [173]. With the dire condition of As-contaminated drinking water in mind, numerous strategies have been investigated, including physicochemical methods (sorption, ion exchange, precipitation, coagulation, membrane filtration, and permeable reactive barriers) and biological processes (phytoremediation, biological treatment with living microbes). Sorption has appeared as a cost-effective and environmentally friendly option for heavy metals cleanup among these approaches. Biosorption is the method of eliminating heavy metals such as As utilizing biosorbents. Biosorption is a process in which a non-living (inactive) biomass binds to and removes As from water via physicochemical reactions (e.g., chelation, adsorption, precipitation) [174]. These approaches are expensive, induce soil disturbance, and often introduce secondary contaminants, all of which alter the natural mechanism of soil ecosystems.

### 4.5. Phytoremediation

**Figure 5:**
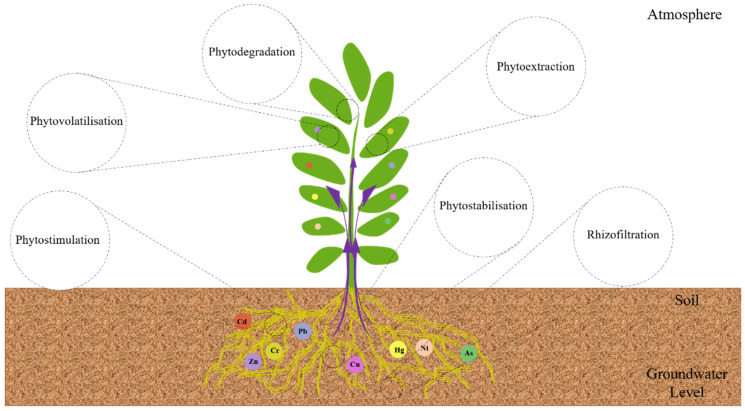
The mechanisms by which plants absorb heavy metals via phytoremediation technology.

Several chemical and physical remediation strategies have been utilized, but now scientists have increasingly turned their attention to biological methods for heavy metal remediation, such as “Phytoremediation” is a word made up of two words: Greek words: Greek phyto (meaning plant) and Latin remedium (meaning remedy) [2]. Plants are innately capable of absorbing inorganic compounds (including metals) from the soil and sediment [86]. Chelates solubilize heavy metals to make them available for uptake; however, they are not always available to plants [175]. Hyperaccumulator plants are vital due to their extraordinary ability to absorb, convert, and accumulate elevated levels of Cd/Pb without phytotoxicity [176]. More than 200 plant species have been recognized as hyperaccumulators of heavy metals, although their biomass output is too low for these plants to be viable remediation candidates in the soil [177]. To absorb various organic and inorganic pollutants, plants use several approaches, including phytoextraction, phytostimulation, phytostabilization, phytodegradation, phytovolatilization, and rhizofiltration, which form the basis of the phytoremediation technique (Figure 5) [178].

#### 4.5.1. Phytostabilization

Phytostabilization (phytorestoration, or phytoimmobilization) is a process in which plants excrete components to decrease the pH of the soil and produce metal complexes. Plants must be kept separate from wildlife and agricultural lands [70]. The main objective is to keep pollutants from mobilizing and minimize their soluble form while also preventing them from diffusing into the soil. By excreting specialized redox enzymes, plants can transform potentially dangerous metals to a less toxic condition, minimizing metal stress and damage [2]. For instance, Cr(VI) can be oxidized to form Cr(III), a less mobile and hazardous form [70]. Nevertheless, phytostabilization is not a long-lasting solution because the heavy metals persist in the soil [161], only their mobility is reduced [2]. Utilization of plants to decrease the mobility and bioavailability of contaminants in the environment, hence avoiding their migration into groundwater and the food chain [179] and sequestering the pollutants in or on cell wall lignins (lignification), absorption of pollutants by soil humus via plant or microbial enzymes (humification), or other methods by which the pollutant is sequestered in the soil, for example, through binding to organic matter [180].

#### 4.5.2. Phytostimulation

Phytostimulation (also called rhizoremediation, rhizodegradation, microbe-assisted phytoremediation) enhances soil microbial activity to help in pollution degradation. In several circumstances, rhizosphere bacteria have been discovered to be substantial contributors to the pollution breakdown process. Because of environmental restrictors present at locations such as weed competition, restricted plant evolution in a contaminated environment, the appearance of plant infections, and other abiotic/biotic stressors, the microbial augmented rhizoremediation process is substantially slower than ex-situ operations [178]. The release of exudates/enzymes into the root zone (rhizosphere) stimulates microbial and fungal breakdown in plant-assisted bioremediation [179].

#### 4.5.3. Phytovolatilization

With phytovolatilization, heavy metal contaminants can be removed from the location and dispersed as gaseous molecules without harvesting and disposing plants like other phytoremediation methods. But, its application is constrained because it does not remove the contaminant; instead, it transports it from one phase (soil) to another (air), where it could be redeposited [2]. These are the advantages of phytovolatilization: For example, pollutants such as elemental mercury and dimethyl selenite gas might be converted into less harmful forms, whereas pollutants or their metabolites are released into the atmosphere be exposed to more efficient or fast natural degradation processes such as photodegradation. Mercury and selenium, for example, can be transformed into harmless forms and released into the air by plants’ roots, shoots, or leaves after the roots have taken them up [180].

#### 4.5.4. Rhizofiltration

Rhizofiltration is described as removing contaminants from surface water, wastewater, or groundwater extracted to the surface by plant roots through adsorption, precipitation, or absorption [181]. A plant suitable for rhizofiltration should have a rapid root growth approach and the capacity to remove metals from the solution over an extended time [178]. Plant species, groundwater conditions (temperature and pH), and chemical properties of organic pollutants impact rhizofiltration efficiency [182].

#### 4.5.5. Phytoextraction

Phytoextraction (also recognized as phytoaccumulation, phytosequestration, or phytoabsorption), the ability of plants to absorb inorganic (particularly metal) pollutants from soil, is becoming a more extensively utilized remediation technique [86]. Phytoextraction utilizes rapidly growing plants to remove heavy metals from the environment (soil and water) [161]. Because harvesting root biomass is often not feasible, metal transfer to shoots is an important biochemical process [2]. Compared to current remediation methods, this developing green technology would be ten times more cost-effective [161] and uses hyperaccumulator plants [176]. While polluted soils frequently contain high concentrations of many toxic trace elements, drastic improvements are needed for phytoextraction to become a viable method, i.e., plants with high tolerance and accumulation rates for several metals [180].

#### 4.5.6. Phytodegradation

Phytodegradation (also called phytotransformation) is the process by which organic pollutants are consumed and degraded within the metabolic capabilities of plants and their associated microorganisms, or the fragmentation of organic pollutants in soil, sludge, surface water, or groundwater using enzymes such as dehalogenase, peroxidase, nitroreductase, laccase, and nitrilase [178]. Organic xenobiotics can be accumulated by plants from contaminated surroundings and detoxified through their metabolic processes [2]. It is known that plants produce enzymatic substances like dehalogenases and oxygenases. Phytovolatilization refers to the discharge of volatile substances into the air due to plant transpiration. These plants collect pollutants from the soil, convert them into volatile compounds, and release them into the atmosphere [161]. The various phytoremediation techniques show that phytoextraction, rhizofiltration, and phytostabilization methods offer prospective commercial entrepreneurship opportunities in the environmental remediation industry [180].

## 5. Future Scope

However, in recent years, the problem of heavy metals in the environment has become prominent, coupled with regional differences, resulting in the difficulty of pollution control. In order to achieve a harmonious and long-term development, it is necessary to strengthen the management of soil pollution, especially heavy metal pollution, strengthen the restoration of soil, improve the environment for human survival, and create comfortable environmental conditions for future generations. Soil heavy metal pollution and mechanisms will be carefully analyzed in the future to test the source of heavy metal pollution. Soil heavy metal detection technology still has a lot of potential for improvement. In the future, the soil heavy metal detection technology will be intelligent, integration, high precision, low cost, support; this is another shell realization of the concept of green development. The future development trend of soil metal detection technology must be highly automated. At the same time, it is necessary to establish and improve laws and regulations to control the discharge of mining and smelting industries, raise people’s awareness of environmental protection, and promote the prevention and control of soil pollution, thereby restoring people’s health and ensuring sustainable ecological development. In recent years, with the accelerated urbanization process in China, mineral resources, sewage irrigation, and an unreasonable application of chemical fertilizers and pesticides have led to the continuous accumulation of heavy metals in the soil. The goal of development should be to realize the junction of geology, chemistry, and biology, to deepen theoretical research on the bioavailability of heavy metals, and to build a sub-section index of the bioavailability of soil heavy metal pollution in order to standardize and unify the evaluation technique and to achieve the soil pollution prevention. It’s possible to employ isotopes to correctly identify the source of heavy metals in the environment and understand the involved biogeochemical processes. To recover metal, hydrometallurgy is the leading technology used. Some environmental challenges that need to be addressed in these technologies include effluent, residue, and exhaust gas for recycling minimizing secondary contamination in soil. It is still impossible to accurately identify the amount and concentration. The investigation system will be further improved and standardized the soil restoration market behavior system.

## 6. Conclusions

Soils surrounding mining and smelting areas are often highly contaminated by heavy metals. In China, the main sources of heavy metals contamination are man-made sources, such as industrial contamination from mining and smelting. Heavy metal pollution in the soil is a main source of worry due to its possible influence on animal and human health. Since industrialization and technological development emerged, heavy metals poisoning the soil has been a significant concern in China. Furthermore, soil heavy metal pollution poses significant carcinogenic and non-carcinogenic threats to the community, particularly to kids and those in highly contaminated areas. According to limited research, local children have elevated blood Pb levels (BLLs), which is linked to the detrimental impacts of Pb smelting on inhabitants. Soil pollutants, food safety, and human health are inextricably associated. This review shows the extraction and separation of metals from waste using hydrometallurgical routes of leaching, solvent extraction, and ion exchange to reduce heavy metal pollution. We evaluated the numerous remediation approaches for extracting heavy metals from the soil. The existing remediation options concentrate on reducing the concentration of heavy metals in soil and the food chain. Hyperaccumulator plants are important due to their remarkable ability to absorb, transform, and accumulate high concentrations of heavy metals without phytotoxicity. It is of note that phytoremediation is an eco-friendly approach. Identifying priority control components can assist authorities in building better effective exposure mitigation and management methods while generating innovative remediation approaches in future research.

## Data Availability

Not applicable.

## References

[1] Spurgeon D.J., Lawlor A., Hooper H.L., Wadsworth R., Svendsen C., Thomas L.D., Ellis J.K., Bundy J.G., Keun H.C., Jarup L. (2011). Outdoor and indoor cadmium distributions near an abandoned smelting works and their relations to human exposure. Environ. Pollut..

[2] Ali H., Khan E., Sajad M.A. (2013). Phytoremediation of heavy metals—Concepts and applications. Chemosphere.

[3] Duffus J.H. (2002). “Heavy metals” a meaningless term? (IUPAC Technical Report). Pure Appl. Chem..

[4] Kemp D.D. (1998). The Environment Dictionary.

[5] Oves M., Khan M.S., Zaidi A., Ahmad E. (2012). Soil contamination, nutritive value, and human health risk assessment of heavy metals: An overview. Toxicity of Heavy Metals to Legumes and Bioremediation.

[6] Li C., Zhou K., Qin W., Tian C., Qi M., Yan X., Han W. (2019). A review on heavy metals contamination in soil: Effects, sources, and remediation techniques. Soil Sediment Contam. Int. J..

[7] Chen H., Teng Y., Lu S., Wang Y., Wang J. (2015). Contamination features and health risk of soil heavy metals in China. Sci. Total Environ..

[8] Li Z., Ma Z., van der Kuijp T.J., Yuan Z., Huang L. (2014). A review of soil heavy metal pollution from mines in China: Pollution and health risk assessment. Sci. Total Environ..

[9] Wu H., Yang F., Li H., Li Q., Zhang F., Ba Y., Cui L., Sun L., Lv T., Wang N. (2020). Heavy metal pollution and health risk assessment of agricultural soil near a smelter in an industrial city in China. Int. J. Environ. Health Res..

[10] Guo X. (2007). Cost of pollution in China: Economic estimates of physical damages. World Bank.

[11] Hou D., O’Connor D., Nathanail P., Tian L., Ma Y. (2017). Integrated GIS and multivariate statistical analysis for regional scale assessment of heavy metal soil contamination: A critical review. Environ. Pollut..

[12] Solgi E., Esmaili-Sari A., Riyahi-Bakhtiari A., Hadipour M. (2012). Soil contamination of metals in the three industrial estates, Arak, Iran. Bull. Environ. Contam. Toxicol..

[13] Burges A., Epelde L., Garbisu C. (2015). Impact of repeated single-metal and multi-metal pollution events on soil quality. Chemosphere.

[14] Zhang P., Qin C., Hong X., Kang G., Qin M., Yang D., Pang B., Li Y., He J., Dick R.P. (2018). Risk assessment and source analysis of soil heavy metal pollution from lower reaches of Yellow River irrigation in China. Sci. Total Environ..

[15] Giller K., McGrath S. (1988). Pollution by toxic metals on agricultural soils. Nature.

[16] Rodrigues S., Cruz N., Coelho C., Henriques B., Carvalho L., Duarte A., Pereira E., Römkens P.F. (2013). Risk assessment for Cd, Cu, Pb and Zn in urban soils: Chemical availability as the central concept. Environ. Pollut..

[17] Zhou J., Liang J., Hu Y., Zhang W., Liu H., You L., Zhang W., Gao M., Zhou J. (2018). Exposure risk of local residents to copper near the largest flash copper smelter in China. Sci. Total Environ..

[18] Shi T., Ma J., Zhang Y., Liu C., Hu Y., Gong Y., Wu X., Ju T., Hou H., Zhao L. (2019). Status of lead accumulation in agricultural soils across China (1979–2016). Environ. Int..

[19] Zhao F.-J., Ma Y., Zhu Y.-G., Tang Z., McGrath S.P. (2015). Soil contamination in China: Current status and mitigation strategies. Environ. Sci. Technol..

[20] Adnan M., Xiao B., Xiao P., Zhao P., Bibi S. (2022). Heavy Metal, Waste, COVID-19, and Rapid Industrialization in This Modern Era—Fit for Sustainable Future. Sustainability.

[21] Mielke H.W., Reagan P.L. (1998). Soil is an important pathway of human lead exposure. Environ. Health Perspect..

[22] Wang S., Zhang J. (2006). Blood lead levels in children, China. Environ. Res..

[23] Qiu K., Xing W., Scheckel K., Cheng Y., Zhao Z., Ruan X., Li L. (2016). Temporal and seasonal variations of As, Cd and Pb atmospheric deposition flux in the vicinity of lead smelters in Jiyuan, China. Atmos. Pollut. Res..

[24] Stafilov T., Šajn R., Boev B., Cvetković J., Mukaetov D., Andreevski M., Lepitkova S. (2010). Distribution of some elements in surface soil over the Kavadarci region, Republic of Macedonia. Environ. Earth Sci..

[25] Masindi V., Muedi K.L. (2018). Environmental contamination by heavy metals. Heavy Met..

[26] Wang J., Su J., Li Z., Liu B., Cheng G., Jiang Y., Li Y., Zhou S., Yuan W. (2019). Source apportionment of heavy metal and their health risks in soil-dustfall-plant system nearby a typical non-ferrous metal mining area of Tongling, Eastern China. Environ. Pollut..

[27] Zhang X., Zhong T., Liu L., Ouyang X. (2015). Impact of soil heavy metal pollution on food safety in China. PLoS ONE.

[28] Liu G., Tao L., Liu X., Hou J., Wang A., Li R. (2013). Heavy metal speciation and pollution of agricultural soils along Jishui River in non-ferrous metal mine area in Jiangxi Province, China. J. Geochem. Explor..

[29] Bi X., Zhang M., Wu Y., Fu Z., Sun G., Shang L., Li Z., Wang P. (2020). Distribution patterns and sources of heavy metals in soils from an industry undeveloped city in Southern China. Ecotoxicol. Environ. Saf..

[30] Bermudez G.M., Jasan R., Plá R., Pignata M.L. (2012). Heavy metals and trace elements in atmospheric fall-out: Their relationship with topsoil and wheat element composition. J. Hazard. Mater..

[31] Luo L., Ma Y., Zhang S., Wei D., Zhu Y.-G. (2009). An inventory of trace element inputs to agricultural soils in China. J. Environ. Manag..

[32] Ran Y., Xing W., Liang S., Xiang G., Li L. (2010). Heavy metal availability in soil near a lead smelter in the North China Plain. Asian J. Ecotoxicol..

[33] Li Z., Feng X., Li G., Bi X., Sun G., Zhu J., Qin H., Wang J. (2011). Mercury and other metal and metalloid soil contamination near a Pb/Zn smelter in east Hunan province, China. Appl. Geochem..

[34] Lu H., Li Z., Gasco G., Mendez A., Shen Y., Paz-Ferreiro J. (2018). Use of magnetic biochars for the immobilization of heavy metals in a multi-contaminated soil. Sci. Total Environ..

[35] Wang S.-L., Xu X.-R., Sun Y.-X., Liu J.-L., Li H.-B. (2013). Heavy metal pollution in coastal areas of South China: A review. Mar. Pollut. Bull..

[36] Li L., Zhang Y., Ippolito J.A., Xing W., Qiu K., Yang H. (2020). Lead smelting effects heavy metal concentrations in soils, wheat, and potentially humans. Environ. Pollut..

[37] Williams P., Lei M., Sun G., Huang Q., Lu Y., Deacon C., Meharg A.A., Zhu Y.-G. (2009). Occurrence and partitioning of cadmium, arsenic and lead in mine impacted paddy rice: Hunan, China. Environ. Sci. Technol..

[38] Xie F., Tan H., Yang B., He J.L., Chen A.N., Wen X.M. (2014). The study of atmospheric transport and deposition of cadmium emitted from primitive zinc production area. Water Air Soil Pollut..

[39] Han L., Gao B., Hao H., Zhou H., Lu J., Sun K. (2018). Lead contamination in sediments in the past 20 years: A challenge for China. Sci. Total Environ..

[40] Chen R., De Sherbinin A., Ye C., Shi G. (2014). China’s soil pollution: Farms on the frontline. Science.

[41] Jiang Z., Guo Z., Peng C., Liu X., Zhou Z., Xiao X. (2021). Heavy metals in soils around non-ferrous smelteries in China: Status, health risks and control measures. Environ. Pollut..

[42] Shao X., Huang B., Zhao Y., Sun W., Gu Z., Qian W. (2014). Impacts of human activities and sampling strategies on soil heavy metal distribution in a rapidly developing region of China. Ecotoxicol. Environ. Saf..

[43] Ettler V., Mihaljevič M., Kříbek B., Majer V., Šebek O. (2011). Tracing the spatial distribution and mobility of metal/metalloid contaminants in Oxisols in the vicinity of the Nkana copper smelter, Copperbelt province, Zambia. Geoderma.

[44] Morrison A.L., Gulson B.L. (2007). Preliminary findings of chemistry and bioaccessibility in base metal smelter slags. Sci. Total Environ..

[45] Lee P.-K., Kang M.-J., Yu S., Kwon Y.K. (2020). Assessment of trace metal pollution in roof dusts and soils near a large Zn smelter. Sci. Total Environ..

[46] Du B., Zhou J., Lu B., Zhang C., Li D., Zhou J., Jiao S., Zhao K., Zhang H. (2020). Environmental and human health risks from cadmium exposure near an active lead-zinc mine and a copper smelter, China. Sci. Total Environ..

[47] Yang Q., Li Z., Lu X., Duan Q., Huang L., Bi J. (2018). A review of soil heavy metal pollution from industrial and agricultural regions in China: Pollution and risk assessment. Sci. Total Environ..

[48] Ettler V. (2016). Soil contamination near non-ferrous metal smelters: A review. Appl. Geochem..

[49] Jamal A., Delavar M.A., Naderi A., Nourieh N., Medi B., Mahvi A.H. (2018). Distribution and health risk assessment of heavy metals in soil surrounding a lead and zinc smelting plant in Zanjan, Iran. Hum. Ecol. Risk Assess. Int. J..

[50] Lei K., Giubilato E., Critto A., Pan H., Lin C. (2016). Contamination and human health risk of lead in soils around lead/zinc smelting areas in China. Environ. Sci. Pollut. Res..

[51] Yu L., Wang Y.-b., Xin G., Su Y.-b., Gang W. (2006). Risk assessment of heavy metals in soils and vegetables around non-ferrous metals mining and smelting sites, Baiyin, China. J. Environ. Sci..

[52] Ettler V., Konečný L., Kovářová L., Mihaljevič M., Šebek O., Kříbek B., Majer V., Veselovský F., Penížek V., Vaněk A. (2014). Surprisingly contrasting metal distribution and fractionation patterns in copper smelter-affected tropical soils in forested and grassland areas (Mufulira, Zambian Copperbelt). Sci. Total Environ..

[53] Xing W., Zheng Y., Scheckel K.G., Luo Y., Li L. (2019). Spatial distribution of smelter emission heavy metals on farmland soil. Environ. Monit. Assess..

[54] He B., Wang W., Geng R., Ding Z., Luo D., Qiu J., Zheng G., Fan Q. (2021). Exploring the fate of heavy metals from mining and smelting activities in soil-crop system in Baiyin, NW China. Ecotoxicol. Environ. Saf..

[55] Kang Z., Wang S., Qin J., Wu R., Li H. (2020). Pollution characteristics and ecological risk assessment of heavy metals in paddy fields of Fujian province, China. Sci. Rep..

[56] Briki M., Zhu Y., Gao Y., Shao M., Ding H., Ji H. (2017). Distribution and health risk assessment to heavy metals near smelting and mining areas of Hezhang, China. Environ. Monit. Assess..

[57] Wang L., Jin Y., Weiss D.J., Schleicher N.J., Wilcke W., Wu L., Guo Q., Chen J., O’Connor D., Hou D. (2021). Possible application of stable isotope compositions for the identification of metal sources in soil. J. Hazard. Mater..

[58] Rabinowitz M.B. (2005). Lead isotopes in soils near five historic American lead smelters and refineries. Sci. Total Environ..

[59] Zhu G., Guo Q., Xiao H., Chen T., Yang J. (2017). Multivariate statistical and lead isotopic analyses approach to identify heavy metal sources in topsoil from the industrial zone of Beijing Capital Iron and Steel Factory. Environ. Sci. Pollut. Res..

[60] Pratte S., Bao K., Shen J., Mackenzie L., Klamt A.-M., Wang G., Xing W. (2018). Recent atmospheric metal deposition in peatlands of northeast China: A review. Sci. Total Environ..

[61] Kaste J.M., Friedland A.J., Stürup S. (2003). Using stable and radioactive isotopes to trace atmospherically deposited Pb in montane forest soils. Environ. Sci. Technol..

[62] Ma Y., Song X. (2016). Using stable isotopes to determine seasonal variations in water uptake of summer maize under different fertilization treatments. Sci. Total Environ..

[63] Zhou J., Obrist D., Dastoor A., Jiskra M., Ryjkov A. (2021). Vegetation uptake of mercury and impacts on global cycling. Nat. Rev. Earth Environ..

[64] Bigalke M., Weyer S., Kobza J., Wilcke W. (2010). Stable Cu and Zn isotope ratios as tracers of sources and transport of Cu and Zn in contaminated soil. Geochim. Cosmochim. Acta.

[65] Ellis A.S., Johnson T.M., Bullen T.D. (2002). Chromium isotopes and the fate of hexavalent chromium in the environment. Science.

[66] Maréchal C.N., Télouk P., Albarède F. (1999). Precise analysis of copper and zinc isotopic compositions by plasma-source mass spectrometry. Chem. Geol..

[67] Vaněk A., Vejvodová K., Mihaljevič M., Ettler V., Trubač J., Vaňková M., Teper L., Cabala J., Sutkowska K., Voegelin A. (2022). Evaluation of thallium isotopic fractionation during the metallurgical processing of sulfides: An update. J. Hazard. Mater..

[68] Ventura G.T., Gall L., Siebert C., Prytulak J., Szatmari P., Hurlimann M., Halliday A.N. (2015). The stable isotope composition of vanadium, nickel, and molybdenum in crude oils. Appl. Geochem..

[69] Šillerová H., Chrastný V., Vítková M., Francová A., Jehlička J., Gutsch M.R., Kocourková J., Aspholm P.E., Nilsson L.O., Berglen T.F. (2017). Stable isotope tracing of Ni and Cu pollution in North-East Norway: Potentials and drawbacks. Environ. Pollut..

[70] Mulligan C., Yong R., Gibbs B. (2001). Remediation technologies for metal-contaminated soils and groundwater: An evaluation. Eng. Geol..

[71] Liu R., Wang M., Chen W., Peng C. (2016). Spatial pattern of heavy metals accumulation risk in urban soils of Beijing and its influencing factors. Environ. Pollut..

[72] Di X., Xiao B., Dong H., Wang S. (2019). Implication of different humic acid fractions in soils under karst rocky desertification. Catena.

[73] Tang H., Xiao B., Xiao P. (2021). Interaction of Ca2+ and soil humic acid characterized by a joint experimental platform of potentiometric titration, UV–Visible spectroscopy, and fluorescence spectroscopy. Acta Geochim..

[74] Chrastný V., Vanek A., Teper L., Cabala J., Procházka J., Pechar L., Drahota P., Penížek V., Komárek M., Novák M. (2012). Geochemical position of Pb, Zn and Cd in soils near the Olkusz mine/smelter, South Poland: Effects of land use, type of contamination and distance from pollution source. Environ. Monit. Assess..

[75] Bakshi S., Banik C., He Z. (2018). The impact of heavy metal contamination on soil health. Manag. Soil Health Sustain. Agric..

[76] Jacob J.M., Karthik C., Saratale R.G., Kumar S.S., Prabakar D., Kadirvelu K., Pugazhendhi A. (2018). Biological approaches to tackle heavy metal pollution: A survey of literature. J. Environ. Manag..

[77] Song J., Huang G., Han D., Hou Q., Gan L., Zhang M. (2021). A review of reactive media within permeable reactive barriers for the removal of heavy metal (loid) s in groundwater: Current status and future prospects. J. Clean. Prod..

[78] Qasem N.A., Mohammed R.H., Lawal D.U. (2021). Removal of heavy metal ions from wastewater: A comprehensive and critical review. Npj Clean Water.

[79] Miranda L.S., Ayoko G.A., Egodawatta P., Goonetilleke A. (2022). Adsorption-desorption behavior of heavy metals in aquatic environments: Influence of sediment, water and metal ionic properties. J. Hazard. Mater..

[80] Ji C., Wu D., Lu J., Shan C., Ren Y., Li T., Lv L., Pan B., Zhang W. (2021). Temperature regulated adsorption and desorption of heavy metals to A-MIL-121: Mechanisms and the role of exchangeable protons. Water Res..

[81] Qu J., Fan M. (2010). The current state of water quality and technology development for water pollution control in China. Crit. Rev. Environ. Sci. Technol..

[82] Liu R., Guan Y., Chen L., Lian B. (2018). Adsorption and desorption characteristics of Cd^2+^ and Pb^2+^ by micro and nano-sized biogenic CaCO_3_. Front. Microbiol..

[83] Larson C. (2014). China gets serious about its pollutant-laden soil. Am. Assoc. Adv. Sci..

[84] Karami E., Kuhar L., Bona A., Nikoloski A.N. (2021). A review of electrokinetic, ultrasonic and solution pulsing methods for mass transfer enhancement in in-situ processing. Miner. Eng..

[85] Varol M. (2011). Assessment of heavy metal contamination in sediments of the Tigris River (Turkey) using pollution indices and multivariate statistical techniques. J. Hazard. Mater..

[86] Arthur E.L., Rice P.J., Rice P.J., Anderson T.A., Baladi S.M., Henderson K.L., Coats J.R. (2005). Phytoremediation—an overview. Crit. Rev. Plant Sci..

[87] Morin G., Ostergren J.D., Juillot F., Ildefonse P., Calas G., Brown G.E. (1999). XAFS determination of the chemical form of lead in smelter-contaminated soils and mine tailings: Importance of adsorption processes. Am. Mineral..

[88] Popescu I., Nimirciag R., Vina G., Ajmone-Marsan F. (2013). Spatial distribution of potentially toxic elements in soils polluted by mining activities. Rev. Chim..

[89] Popescu I., Stanescu R., Biasioli M., Ajmone Marsan F., Constantinescu I. (2013). Assessing human risks through CSOIL exposure model for a soil contamination associated to heavy metals. Univ. Politeh. Buchar. Sci. Bull. Ser. B.

[90] Karna R.R., Noerpel M., Betts A.R., Scheckel K.G. (2017). Lead and arsenic bioaccessibility and speciation as a function of soil particle size. J. Environ. Qual..

[91] Zou M., Zhou S., Zhou Y., Jia Z., Guo T., Wang J. (2021). Cadmium pollution of soil-rice ecosystems in rice cultivation dominated regions in China: A review. Environ. Pollut..

[92] Nag R., O’Rourke S.M., Cummins E. (2022). Risk factors and assessment strategies for the evaluation of human or environmental risk from metal (loid) s–A focus on Ireland. Sci. Total Environ..

[93] Ruby M.V., Schoof R., Brattin W., Goldade M., Post G., Harnois M., Mosby D.E., Casteel S.W., Berti W., Carpenter M. (1999). Advances in evaluating the oral bioavailability of inorganics in soil for use in human health risk assessment. Environ. Sci. Technol..

[94] Rieuwerts J.S., Farago M.E. (1995). Lead contamination in smelting and mining environments and variations in chemical forms and bioavailability. Chem. Speciat. Bioavailab..

[95] Xiao X., Zhang J., Wang H., Han X., Ma J., Ma Y., Luan H. (2020). Distribution and health risk assessment of potentially toxic elements in soils around coal industrial areas: A global meta-analysis. Sci. Total Environ..

[96] Muller G. (1969). Index of geoaccumulation in sediments of the Rhine River. Geojournal.

[97] Yaqin J., Yinchang F., Jianhui W., Tan Z., Zhipeng B., Chiqing D. (2008). Using geoaccumulation index to study source profiles of soil dust in China. J. Environ. Sci..

[98] Cai L.-M., Wang Q.-S., Wen H.-H., Luo J., Wang S. (2019). Heavy metals in agricultural soils from a typical township in Guangdong Province, China: Occurrences and spatial distribution. Ecotoxicol. Environ. Saf..

[99] Wang J., Zhang X., Yang Q., Zhang K., Zheng Y., Zhou G. (2018). Pollution characteristics of atmospheric dustfall and heavy metals in a typical inland heavy industry city in China. J. Environ. Sci..

[100] Kabata-Pendias A. (2011). Trace Elements in Soil and Plants.

[101] Lu X., Wang L., Lei K., Huang J., Zhai Y. (2009). Contamination assessment of copper, lead, zinc, manganese and nickel in street dust of Baoji, NW China. J. Hazard. Mater..

[102] Šajn R., Aliu M., Stafilov T., Alijagić J. (2013). Heavy metal contamination of topsoil around a lead and zinc smelter in Kosovska Mitrovica/Mitrovicë, Kosovo/Kosovë. J. Geochem. Explor..

[103] Xu D.-M., Fu R.-B., Liu H.-Q., Guo X.-P. (2021). Current knowledge from heavy metal pollution in Chinese smelter contaminated soils, health risk implications and associated remediation progress in recent decades: A critical review. J. Clean. Prod..

[104] Pérez K., Toro N., Gálvez E., Robles P., Wilson R., Navarra A. (2021). Environmental, economic and technological factors affecting Chilean copper smelters—A critical review. J. Mater. Res. Technol..

[105] Wenzel W.W., Kirchbaumer N., Prohaska T., Stingeder G., Lombi E., Adriano D.C. (2001). Arsenic fractionation in soils using an improved sequential extraction procedure. Anal. Chim. Acta.

[106] Stafilov T., Šajn R., Pančevski Z., Boev B., Frontasyeva M.V., Strelkova L.P. (2010). Heavy metal contamination of topsoils around a lead and zinc smelter in the Republic of Macedonia. J. Hazard. Mater..

[107] He M. (2007). Distribution and phytoavailability of antimony at an antimony mining and smelting area, Hunan, China. Environ. Geochem. Health.

[108] Wu Q., Wang S., Wang L., Liu F., Lin C.-J., Zhang L., Wang F. (2014). Spatial distribution and accumulation of Hg in soil surrounding a Zn/Pb smelter. Sci. Total Environ..

[109] Yin X., Yao C., Song J., Li Z., Zhang C., Qian W., Bi D., Li C., Teng Y., Wu L. (2009). Mercury contamination in vicinity of secondary copper smelters in Fuyang, Zhejiang Province, China: Levels and contamination in topsoils. Environ. Pollut..

[110] Feng X., Li G., Qiu G. (2006). A preliminary study on mercury contamination to the environment from artisanal zinc smelting using indigenous methods in Hezhang County, Guizhou, China: Part 2. Mercury contaminations to soil and crop. Sci. Total Environ..

[111] Zhang Z., Zhang N., Li H., Lu Y., Yang Z. (2020). Potential health risk assessment for inhabitants posed by heavy metals in rice in Zijiang River basin, Hunan Province, China. Environ. Sci. Pollut. Res..

[112] Xing W., Cao E., Scheckel K.G., Bai X., Li L. (2018). Influence of phosphate amendment and zinc foliar application on heavy metal accumulation in wheat and on soil extractability impacted by a lead smelter near Jiyuan, China. Environ. Sci. Pollut. Res..

[113] Li Z., Feng X., Bi X., Li G., Lin Y., Sun G. (2014). Probing the distribution and contamination levels of 10 trace metal/metalloids in soils near a Pb/Zn smelter in Middle China. Environ. Sci. Pollut. Res..

[114] Liu L., Wu L., Luo Y., Zhang C., Jiang Y., Qiu X. (2010). The impact of a copper smelter on adjacent soil zinc and cadmium fractions and soil organic carbon. J. Soils Sediments.

[115] Wang Y., Shi J., Wang H., Lin Q., Chen X., Chen Y. (2007). The influence of soil heavy metals pollution on soil microbial biomass, enzyme activity, and community composition near a copper smelter. Ecotoxicol. Environ. Saf..

[116] Yang Y., Li S., Bi X., Wu P., Liu T., Li F., Liu C. (2010). Lead, Zn, and Cd in slags, stream sediments, and soils in an abandoned Zn smelting region, southwest of China, and Pb and S isotopes as source tracers. J. Soils Sediments.

[117] Cai L.-M., Wang Q.-S., Luo J., Chen L.-G., Zhu R.-L., Wang S., Tang C.-H. (2019). Heavy metal contamination and health risk assessment for children near a large Cu-smelter in central China. Sci. Total Environ..

[118] Cui Y.-J., Zhu Y.-G., Zhai R.-H., Chen D.-Y., Huang Y.-Z., Qiu Y., Liang J.-Z. (2004). Transfer of metals from soil to vegetables in an area near a smelter in Nanning, China. Environ. Int..

[119] Girault F., Perrier F., Poitou C., Isambert A., Théveniaut H., Laperche V., Clozel-Leloup B., Douay F. (2016). Effective radium concentration in topsoils contaminated by lead and zinc smelters. Sci. Total Environ..

[120] Yang X., Yang Y., Wan Y., Wu R., Feng D., Li K. (2021). Source identification and comprehensive apportionment of the accumulation of soil heavy metals by integrating pollution landscapes, pathways, and receptors. Sci. Total Environ..

[121] Cheng Y., Zhao Z., Wang Y., Qiu K., Fu Y., Zhao X., Li L. (2014). Soil heavy metal pollution in the farmland near a lead-smelter in Henan Province. Chin. J. Soil Sci..

[122] Sundaray S.K., Nayak B.B., Lin S., Bhatta D. (2011). Geochemical speciation and risk assessment of heavy metals in the river estuarine sediments—A case study: Mahanadi basin, India. J. Hazard. Mater..

[123] Pan L., Wang Y., Ma J., Hu Y., Su B., Fang G., Wang L., Xiang B. (2018). A review of heavy metal pollution levels and health risk assessment of urban soils in Chinese cities. Environ. Sci. Pollut. Res..

[124] Jiang J., Khan A.U., Shi B., Tang S., Khan J. (2019). Application of positive matrix factorization to identify potential sources of water quality deterioration of Huaihe River, China. Appl. Water Sci..

[125] Fei J.-C., Min X.-B., Wang Z.-X., Pang Z.-h., Liang Y.-J., Ke Y. (2017). Health and ecological risk assessment of heavy metals pollution in an antimony mining region: A case study from South China. Environ. Sci. Pollut. Res..

[126] Qing X., Yutong Z., Shenggao L. (2015). Assessment of heavy metal pollution and human health risk in urban soils of steel industrial city (Anshan), Liaoning, Northeast China. Ecotoxicol. Environ. Saf..

[127] Liu G., Wang J., Liu X., Liu X., Li X., Ren Y., Wang J., Dong L. (2018). Partitioning and geochemical fractions of heavy metals from geogenic and anthropogenic sources in various soil particle size fractions. Geoderma.

[128] Zhang Z., Lu Y., Li H., Tu Y., Liu B., Yang Z. (2018). Assessment of heavy metal contamination, distribution and source identification in the sediments from the Zijiang River, China. Sci. Total Environ..

[129] Quan Z., Huang W., Liao Y., Liu W., Xu H., Yan N., Qu Z. (2019). Study on the regenerable sulfur-resistant sorbent for mercury removal from nonferrous metal smelting flue gas. Fuel.

[130] Cheng H., Li M., Zhao C., Li K., Peng M., Qin A., Cheng X. (2014). Overview of trace metals in the urban soil of 31 metropolises in China. J. Geochem. Explor..

[131] Shao X., Cheng H., Li Q., Lin C. (2013). Anthropogenic atmospheric emissions of cadmium in China. Atmos. Environ..

[132] Cheng S. (2003). Heavy metal pollution in China: Origin, pattern and control. Environ. Sci. Pollut. Res..

[133] Guo B., Su Y., Pei L., Wang X., Wei X., Zhang B., Zhang D., Wang X. (2020). Contamination, Distribution and Health Risk Assessment of Risk Elements in Topsoil for Amusement Parks in Xi’an, China. Pol. J. Environ. Stud..

[134] Jo I., Koh M. (2004). Chemical changes in agricultural soils of Korea: Data review and suggested countermeasures. Environ. Geochem. Health.

[135] Sow A.Y., Ismail A., Zulkifli S.Z., Amal M.N., Hambali K.A. (2019). Survey on heavy metals contamination and health risk assessment in commercially valuable Asian swamp eel, Monopterus albus from Kelantan, Malaysia. Sci. Rep..

[136] Li M.-M., Gao Z.-Y., Dong C., Wu M.-Q., Yan J., Cao J., Ma W.-J., Wang J., Gong Y.-L., Xu J. (2020). Contemporary blood lead levels of children aged 0–84 months in China: A national cross-sectional study. Environ. Int..

[137] Liu Y., Liu F., Dong K.F., Wu Y., Yang X., Yang J., Tan H., Niu X., Zhao X., Xiao G. (2021). Regional characteristics of children’s blood lead levels in China: A systematic synthesis of national and subnational population data. Sci. Total Environ..

[138] Ji A., Wang F., Luo W., Yang R., Chen J., Cai T. (2011). Lead poisoning in China: A nightmare from industrialisation. Lancet.

[139] Wang H.-Z., Cai L.-M., Wang Q.-S., Hu G.-C., Chen L.-G. (2021). A comprehensive exploration of risk assessment and source quantification of potentially toxic elements in road dust: A case study from a large Cu smelter in central China. Catena.

[140] Kandra P., Pavuluri H., Grandhi S.K., Kandula V.N., Challa L. (2021). Current Trends and Future Perspectives of Biobased Methods for Recovery of Metals from WEEE for a Sustainable Environment. Environmental Management of Waste Electrical and Electronic Equipment.

[141] Gunarathne V., Rajapaksha A.U., Vithanage M., Alessi D.S., Selvasembian R., Naushad M., You S., Oleszczuk P., Ok Y.S. (2020). Hydrometallurgical processes for heavy metals recovery from industrial sludges. Crit. Rev. Environ. Sci. Technol..

[142] Binnemans K., Jones P.T., Fernández Á.M., Torres V.M. (2020). Hydrometallurgical processes for the recovery of metals from steel industry by-products: A critical review. J. Sustain. Metall..

[143] Jadhav U., Hocheng H. (2015). Hydrometallurgical recovery of metals from large printed circuit board pieces. Sci. Rep..

[144] Zeng X., Yang C., Chiang J.F., Li J. (2017). Innovating e-waste management: From macroscopic to microscopic scales. Sci. Total Environ..

[145] Gupta C.K. (2006). Chemical Metallurgy: Principles and Practice.

[146] Vestola E.A., Kuusenaho M.K., Närhi H.M., Tuovinen O.H., Puhakka J.A., Plumb J.J., Kaksonen A. (2010). Acid bioleaching of solid waste materials from copper, steel and recycling industries. Hydrometallurgy.

[147] Yong Y.S., Lim Y.A., Ilankoon I. (2019). An analysis of electronic waste management strategies and recycling operations in Malaysia: Challenges and future prospects. J. Clean. Prod..

[148] Wu P., Zhang L.-J., Lin C.-B., Xie X.-X., Yong X.-Y., Wu X.-Y., Zhou J., Jia H.-H., Wei P. (2020). Extracting heavy metals from electroplating sludge by acid and bioelectrical leaching using Acidithiobacillus ferrooxidans. Hydrometallurgy.

[149] Sethurajan M., van Hullebusch E.D., Nancharaiah Y.V. (2018). Biotechnology in the management and resource recovery from metal bearing solid wastes: Recent advances. J. Environ. Manag..

[150] Li C., Xie F., Ma Y., Cai T., Li H., Huang Z., Yuan G. (2010). Multiple heavy metals extraction and recovery from hazardous electroplating sludge waste via ultrasonically enhanced two-stage acid leaching. J. Hazard. Mater..

[151] Jha M.K., Kumar V., Singh R. (2001). Review of hydrometallurgical recovery of zinc from industrial wastes. Resour. Conserv. Recycl..

[152] Hannula P.-M., Khalid M.K., Janas D., Yliniemi K., Lundström M. (2019). Energy efficient copper electrowinning and direct deposition on carbon nanotube film from industrial wastewaters. J. Clean. Prod..

[153] Trellu C., Mousset E., Pechaud Y., Huguenot D., van Hullebusch E.D., Esposito G., Oturan M.A. (2016). Removal of hydrophobic organic pollutants from soil washing/flushing solutions: A critical review. J. Hazard. Mater..

[154] Jensen J.K., Holm P.E., Nejrup J., Larsen M.B., Borggaard O.K. (2009). The potential of willow for remediation of heavy metal polluted calcareous urban soils. Environ. Pollut..

[155] He C., Zhao Y., Wang F., Oh K., Zhao Z., Wu C., Zhang X., Chen X., Liu X. (2020). Phytoremediation of soil heavy metals (Cd and Zn) by castor seedlings: Tolerance, accumulation and subcellular distribution. Chemosphere.

[156] Liu Y.-N., Xiao X.-Y., Guo Z.-H. (2019). Identification of indicators of giant reed (*Arundo donax* L.) ecotypes for phytoremediation of metal-contaminated soil in a non-ferrous mining and smelting area in southern China. Ecol. Indic..

[157] Fu P., Yang H., Zhang G., Fu P., Li Z. (2019). In-situ immobilization of Cd-contaminated soils using ferronickel slag as potential soil amendment. Bull. Environ. Contam. Toxicol..

[158] Hou D., Al-Tabbaa A. (2014). Sustainability: A new imperative in contaminated land remediation. Environ. Sci. Policy.

[159] Khan F.I., Husain T., Hejazi R. (2004). An overview and analysis of site remediation technologies. J. Environ. Manag..

[160] Liu L., Li W., Song W., Guo M. (2018). Remediation techniques for heavy metal-contaminated soils: Principles and applicability. Sci. Total Environ..

[161] Ashraf S., Ali Q., Zahir Z.A., Ashraf S., Asghar H.N. (2019). Phytoremediation: Environmentally sustainable way for reclamation of heavy metal polluted soils. Ecotoxicol. Environ. Saf..

[162] Chang T., Yen J. (2006). On-site mercury-contaminated soils remediation by using thermal desorption technology. J. Hazard. Mater..

[163] Tran H.T., Lin C., Hoang H.G., Bui X.T., Vu C.T. (2021). Soil washing for the remediation of dioxin-contaminated soil: A Review. J. Hazard. Mater..

[164] Yan D., Guo Z., Xiao X., Peng C., He Y., Yang A., Wang X., Hu Y., Li Z. (2021). Cleanup of arsenic, cadmium, and lead in the soil from a smelting site using N, N-bis (carboxymethyl)-L-glutamic acid combined with ascorbic acid: A lab-scale experiment. J. Environ. Manag..

[165] Mousset E., Oturan M.A., Van Hullebusch E.D., Guibaud G., Esposito G. (2014). Soil washing/flushing treatments of organic pollutants enhanced by cyclodextrins and integrated treatments: State of the art. Crit. Rev. Environ. Sci. Technol..

[166] Vu C.T., Tran H.T., Kaewlaoyoong A., Huang W.-Y., Lin C. (2019). Efficacy of indigenously prepared sugarcane and pineapple wine solvents for washing highly dioxin-contaminated field soils. Appl. Sci..

[167] Makino T., Kamiya T., Takano H., Itou T., Sekiya N., Sasaki K., Maejima Y., Sugahara K. (2007). Remediation of cadmium-contaminated paddy soils by washing with calcium chloride: Verification of on-site washing. Environ. Pollut..

[168] Kim S.-O., Jeong J.Y., Lee W.-C., Yun S.-T., Jo H.Y. (2021). Electrokinetic remediation of heavy metal-contaminated soils: Performance comparison between one-and two-dimensional electrode configurations. J. Soils Sediments.

[169] Tang X., Li Q., Wu M., Lin L., Scholz M. (2016). Review of remediation practices regarding cadmium-enriched farmland soil with particular reference to China. J. Environ. Manag..

[170] Yeung A.T., Hsu C.-N. (2005). Electrokinetic remediation of cadmium-contaminated clay. J. Environ. Eng..

[171] Lu P., Feng Q., Meng Q., Yuan T. (2012). Electrokinetic remediation of chromium-and cadmium-contaminated soil from abandoned industrial site. Sep. Purif. Technol..

[172] Alvarez G.B., Bento N.J.S., Neves T.A., Dos Santos F.S., Silva G.C., de Sousa P.A.P. (2017). Numerical study of the influence of electrode arrangements in electrokinetic remediation technique. Environ. Sci. Pollut. Res..

[173] Khan F.I., Husain T. (2003). Evaluation of a petroleum hydrocarbon contaminated site for natural attenuation using ‘RBMNA’methodology. Environ. Model. Softw..

[174] Shakoor M.B., Niazi N.K., Bibi I., Murtaza G., Kunhikrishnan A., Seshadri B., Shahid M., Ali S., Bolan N.S., Ok Y.S. (2016). Remediation of arsenic-contaminated water using agricultural wastes as biosorbents. Crit. Rev. Environ. Sci. Technol..

[175] Wahla I.H., Kirkham M. (2008). Heavy metal displacement in salt-water-irrigated soil during phytoremediation. Environ. Pollut..

[176] Gul I., Manzoor M., Hashim N., Shah G.M., Waani S.P.T., Shahid M., Antoniadis V., Rinklebe J., Arshad M. (2021). Challenges in microbially and chelate-assisted phytoextraction of cadmium and lead—A review. Environ. Pollut..

[177] Kos B., Leštan D. (2004). Chelator induced phytoextraction and in situ soil washing of Cu. Environ. Pollut..

[178] Kumar V., Shahi S., Singh S. (2018). Bioremediation: An Eco-Sustainable Approach for Restoration of Contaminated Sites. Microbial Bioprospecting for Sustainable Development.

[179] Erakhrumen A.A., Agbontalor A. (2007). Phytoremediation: An environmentally sound technology for pollution prevention, control and remediation in developing countries. Educ. Res. Rev..

[180] Prasad M. (2003). Phytoremediation of metal-polluted ecosystems: Hype for commercialization. Russ. J. Plant Physiol..

[181] Tomé F.V., Rodríguez P.B., Lozano J. (2008). Elimination of natural uranium and 226Ra from contaminated waters by rhizofiltration using *Helianthus annuus* L.. Sci. Total Environ..

[182] Kristanti R.A., Ngu W.J., Yuniarto A., Hadibarata T. (2021). Rhizofiltration for Removal of Inorganic and Organic Pollutants in Groundwater: A Review. Biointerafce Res. Appl. Chem.

